# Insights into the origin of metazoan multicellularity from predatory unicellular relatives of animals

**DOI:** 10.1186/s12915-020-0762-1

**Published:** 2020-04-09

**Authors:** Denis V. Tikhonenkov, Elisabeth Hehenberger, Anton S. Esaulov, Olga I. Belyakova, Yuri A. Mazei, Alexander P. Mylnikov, Patrick J. Keeling

**Affiliations:** 1 Papanin Institute for Biology of Inland Waters, Russian Academy of Sciences, Borok, Russia 152742; 2 Department of Botany, University of British Columbia, Vancouver, British Columbia V6T 1Z4 Canada; 3 Ocean EcoSystems Biology Unit, RD3, GEOMAR Helmholtz Centre for Ocean Research Kiel, Duesternbrookerweg 20, 24105 Kiel, Germany; 4 Penza State University, Penza, Russia 440026; 5 Moscow State University, Moscow, Russia 119991

**Keywords:** Origin of animals, Multicellularity, Protists, Holozoa, *Pigoraptor*, *Syssomonas*

## Abstract

**Background:**

The origin of animals from their unicellular ancestor was one of the most important events in evolutionary history, but the nature and the order of events leading up to the emergence of multicellular animals are still highly uncertain. The diversity and biology of unicellular relatives of animals have strongly informed our understanding of the transition from single-celled organisms to the multicellular Metazoa. Here, we analyze the cellular structures and complex life cycles of the novel unicellular holozoans *Pigoraptor* and *Syssomonas* (Opisthokonta), and their implications for the origin of animals.

**Results:**

*Syssomonas* and *Pigoraptor* are characterized by complex life cycles with a variety of cell types including flagellates, amoeboflagellates, amoeboid non-flagellar cells, and spherical cysts. The life cycles also include the formation of multicellular aggregations and syncytium-like structures, and an unusual diet for single-celled opisthokonts (partial cell fusion and joint sucking of a large eukaryotic prey), all of which provide new insights into the origin of multicellularity in Metazoa. Several existing models explaining the origin of multicellular animals have been put forward, but these data are interestingly consistent with one, the “synzoospore hypothesis.”

**Conclusions:**

The feeding modes of the ancestral metazoan may have been more complex than previously thought, including not only bacterial prey, but also larger eukaryotic cells and organic structures. The ability to feed on large eukaryotic prey could have been a powerful trigger in the formation and development of both aggregative (e.g., joint feeding, which also implies signaling) and clonal (e.g., hypertrophic growth followed by palintomy) multicellular stages that played important roles in the emergence of multicellular animals.

## Background

The origin of animals (Metazoa) from their unicellular ancestors is one of the most important evolutionary transitions in the history of life. Questions about the mechanisms of this transformation arose about 200 years ago, but are still far from being resolved today. Most investigations on the origin of Metazoa have focused on determining the nature of the shared, multicellular ancestor of all contemporary animals [1–3]. However, even the branching order of early, non-bilaterian lineages of animals on phylogenetic trees is still debated: some consider either sponges (Porifera) [4–6] or Ctenophora [7–9] or Placozoa [10, 11] to be the first branch of extant metazoans (although most data show the latter scenario to be the least realistic of these possibilities [2, 12]).

While molecular clock-based studies and paleontological evidence indicate that multicellular animals arose more than 600 million years ago [13, 14], we know very little about how animals arose. To establish the sequence of events in the origin of animals from unicellular ancestors, we also need to investigate their closest relatives, the unicellular opisthokont protists. Information on the diversity and biology of the unicellular relatives of animals, their placement within the phylogenetic tree of opisthokonts, and the identification of molecular and morphological traits thought to be specific for animals within their unicellular sister lineages has all strongly informed our understanding of the transition from single-celled organisms to the multicellular Metazoa [15–19].

Until recently, only three unicellular lineages, the choanoflagellates, filastereans, and ichthyosporeans, as well as *Corallochytrium limacisporum*, a mysterious marine osmotrophic protist described in association with corals, have been described as collectively being sisters to animals. Together with animals, they form the Holozoa within the Opisthokonta [19–21]. These unicellular organisms have extremely variable morphology and biology. Choanoflagellates represent a species-rich group of filter-feeding, bacterivorous, colony-forming protists, which possess a single flagellum surrounded by a collar of tentacles (microvilli). They are subdivided into two main groups—the predominantly marine Acanthoecida and the freshwater and marine Craspedida [22]. Filastereans are amoeboid protists producing pseudopodia. Until recently, they were represented by only two species: the endosymbiont of a freshwater snail, *Capsaspora owczarzaki*, and the free-living marine heterotroph, *Ministeria vibrans* [23, 24], which was recently shown to also possess a single flagellum [19, 25]*.* Ichthyosporeans are parasites or endocommensals of vertebrates and invertebrates characterized by a complex life cycle, reproduction through multinucleated coenocytic colonies, and flagellated and amoeboid dispersal stages [26, 27]. *Corallochytrium* is a unicellular coccoid organism, which produces rough, raised colonies and amoeboid limax-like (slug-shaped) spores [28]. Additionally, molecular data predict a cryptic flagellated stage for *Corallochytrium* [19].

A large number of hypotheses about the origin of multicellular animals have been proposed. The most developed model for the origin of metazoan multicellularity is based on a common ancestor with choanoflagellates [16, 29–33]. This idea was initially based on the observed similarity between choanoflagellates and specialized choanocyte cells in sponges. Molecular investigations also supported the idea by consistently indicating that choanoflagellates are the closest sister group to Metazoa. However, molecular phylogeny itself does not reveal the nature of ancestral states; it only provides a scaffolding on which they might be inferred from other data. The evolutionary positions of the other unicellular holozoans (filastereans, ichthyosporeans, and *Corallochytrium*) are less clear and sometimes controversial (e.g., [19, 24, 34–40]).

As noted above, many molecular traits that were thought to be “animal-specific” are now known to be present in unicellular holozoans, while conversely, the loss of other traits has been shown to correlate with the origin of the animals. But gene content alone is not sufficient to provide a comprehensive understanding of the cell biology, life cycle, and regulation capabilities of the unicellular ancestor; it requires also analysis of the biology of the extant unicellular relatives of animals [41].

**Fig. 1:**
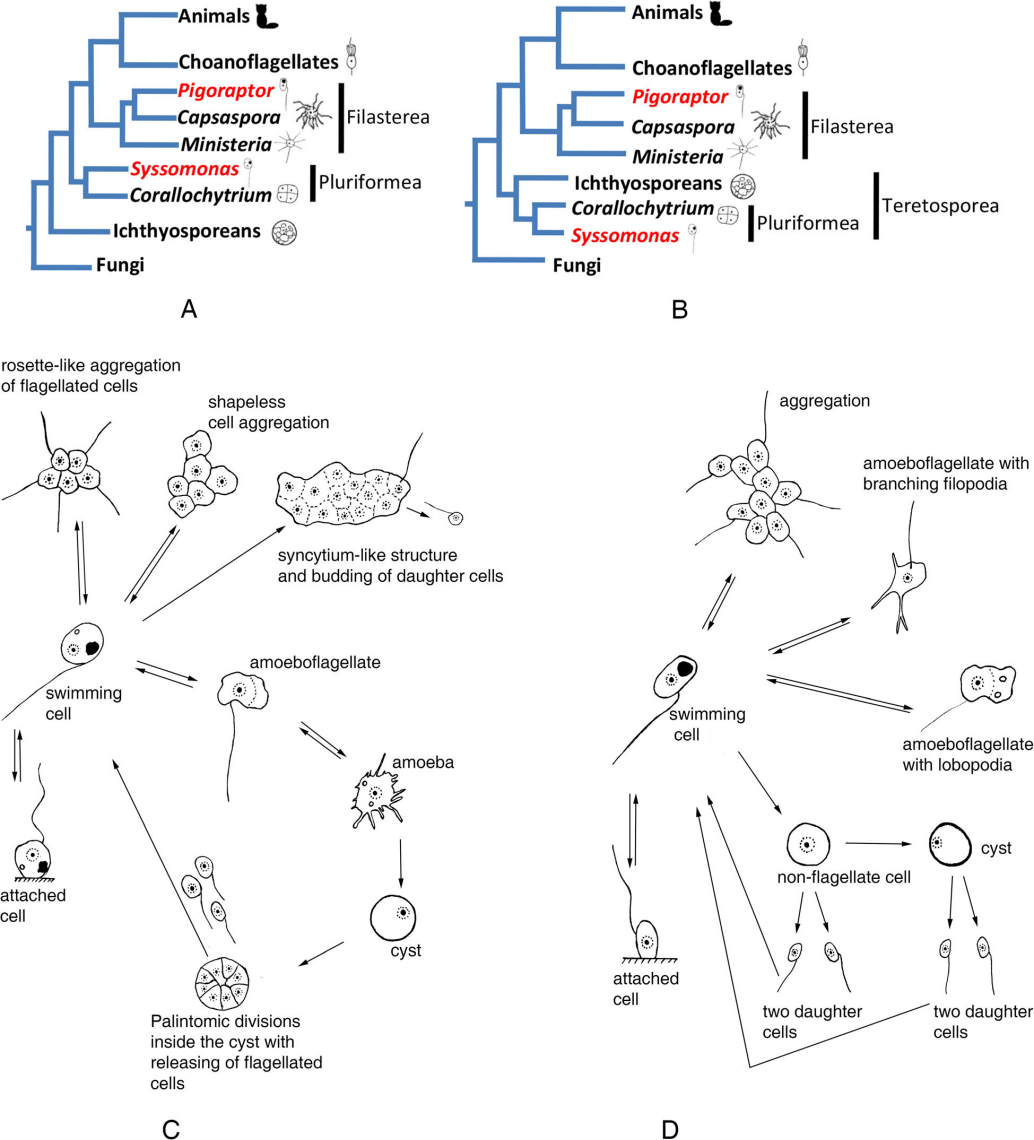
Schemes of the possible phylogenetic position and life cycles of *Syssomonas* and *Pigoraptor*. **a** Pluriformea as a sister lineage to the clade uniting animals, choanoflagellates, and filastereans (including *Pigoraptor*) according to Hehenberger et al. [37]. **b** Pluriformea as a sister lineage to Ichthyosporea within Teretosporea according to López-Escardó et al. [42]. **c** Life cycle of *Syssomonas multiformis*. **d** Life cycle of *Pigoraptor vietnamica* and *P. chileana*

Recently, we described phylogenomic and transciptome analyses of three novel unicellular holozoans [37], which are very similar in morphology and life style but not closely related. *Pigoraptor vietnamica* and *Pigoraptor chileana* are distantly related to filastereans, and *Syssomonas multiformis* forms a new phylogenetic clade, “Pluriformea,” with *Corallochytrium*. The relationship of “Pluriformea” to other holozoans is controversial. Initial analysis indicated “Pluriformea” as a sister lineage to the clade uniting Metazoa, choanoflagellates, and filastereans (Fig. 1a) [37]. Later, single-copy protein domain analysis recovered Pluriformea as sister lineage to Ichthyosporea (Fig. 1b) with almost maximum statistical support [42], validating the Teretosporea hypothesis [19]. Both new genera of unicellular holozoans form the shortest and most slowly evolving branches on the tree, which improved support for many nodes in the phylogeny of unicellular holozoans. Also, comparison of gene content of the new taxa with the known unicellular holozoans revealed several new and interesting distribution patterns for genes related to multicellularity and adhesion [37].

Here, we report the detailed morphological and ultrastructural analyses of these new species, as well as describing their life cycle in culture, which have important implications for understanding the origin of animals as are the genetic analyses. All three species are shown to be predatory flagellates that feed on large eukaryotic prey, which is very unusual for unicellular Holozoa. They also appear to exhibit complex life histories with several distinct stages, including interesting multicellular structures that might offer important clues to precursors of multicellularity. On the basis of these findings, we discuss the current hypotheses about the origin of multicellular animals from their unicellular ancestors.

## Results and discussion

Three novel holozoan taxa were isolated from a freshwater pool (*Syssomonas multiformis*) and the silty sand on the littoral of a freshwater lake (*Pigoraptor vietnamica*) in tropical Vietnam, and from the sediment of a freshwater temporary water body in Tierra del Fuego (*Pigoraptor chileana*). The characteristics of the biotopes are specified in the “Methods” section. Samples were characterized by high species richness of heterotrophic flagellates including bodonids, chrysomonads (*Spumella* spp., *Paraphysomonas* spp.), euglenids (*Petalomonas* spp.), cercomonads, thaumatomonads, protaspids, and loricate bicosoecids. Predatory holozoans appeared to represent a minor fraction of the total abundance. Detailed morphological descriptions of their cells and aggregates are presented below. Note that the term “arrgeration(s)” and cognate words were always used to define a multicellular structure that formed from cells that came together as opposite to the term “clonal multicellularity,” which defines a multicellular structure that formed from a single founding cell that divided repeatedly. All stages of the life cycle (Fig. 1c, d) were observed at 22 °C in the clonal cultures. The main life form in all three studied species is the swimming flagellate cell, which can turn into a cyst, especially in old (~ 1 month) cultures. The amoeboid and pseudopodial stages described below were apparent only after 2 years of cultivation and even then were extremely rare. The variation of temperature and pH, as well as variation of cultivation medium and agitation, did not result in the appearance of additional morphological forms or increase the frequency of occurrence of certain (e.g., amoeboid) life forms. However, increasing the temperature to 30–35 °C leads to suppression and immobilization of prey cells (*Parabodo caudatus*), which favored the feeding of opisthokont predators on slow-moving prey, which in turn lead to an increase in the number of cell aggregations arising from joint feeding.

### *Syssomonas multiformis* morphology and life cycle

**Fig. 2:**
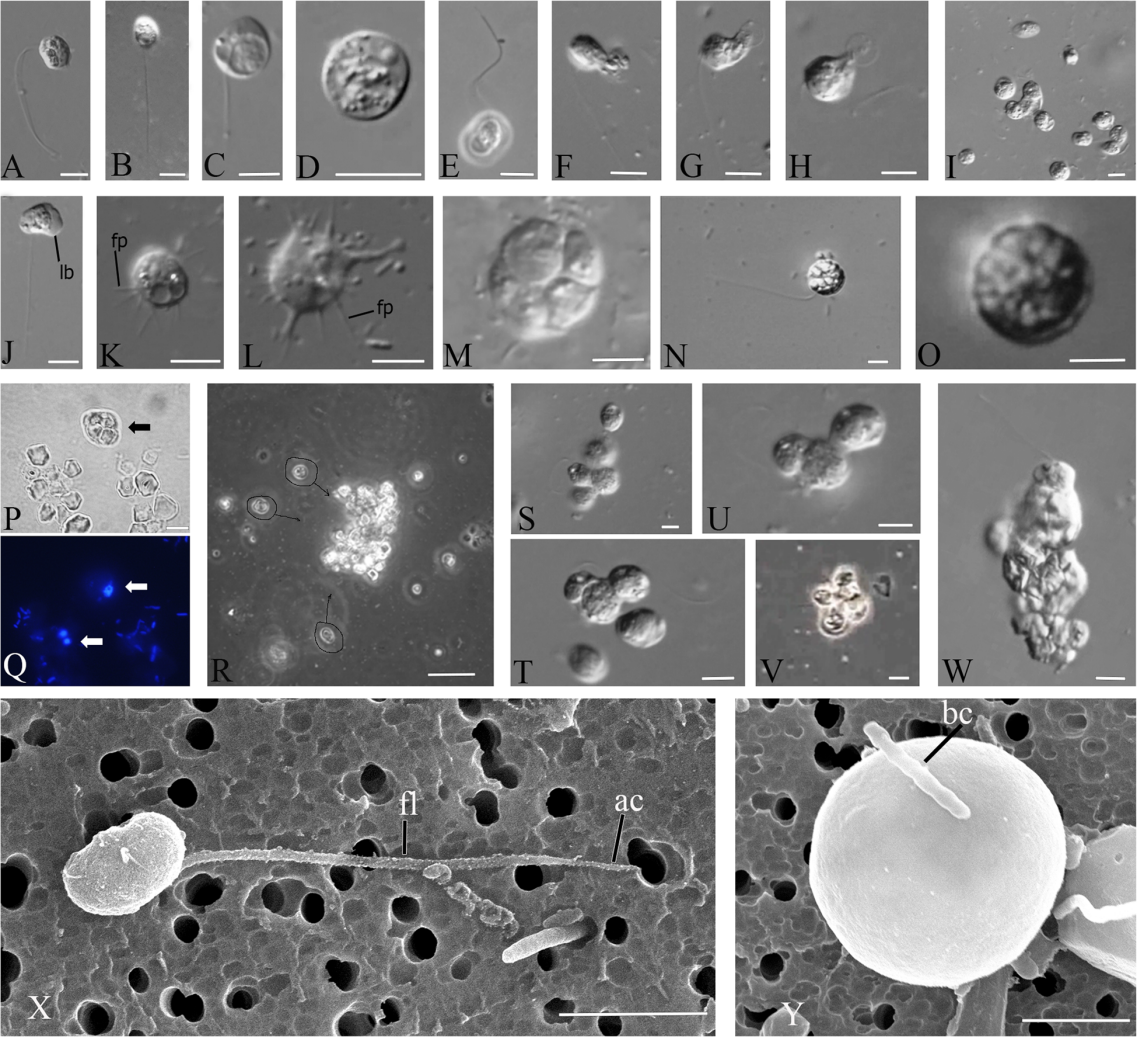
External morphology and life forms of *Syssomonas multiformis*. **a**–**c** Swimming flagellated cells. **d** Cyst. **e** Attached flagellated cell. **f**–**h** Sucking of eukaryotic prey. **i** Simultaneous joint feeding of three cells of *Syssomonas* on one prey cell with attraction of other specimens to the feeding spot. **j** Amoeboflagellate. **k**, **l** Amoeboid cell. **m** Palintomic cell division inside the cyst: the number of observed daughter cells was always even, and up to 16 daughter cells have been seen. **n** Cell with inside vesicles. **o** Cyst with vesicles. **p**, **q** Cells of *Syssomonas* (arrows) with engulfed starch granules (bright field (**p**) and fluorescent microscopy, DAPI staining; arrows are pointing to *Syssomonas* cells). **r** Cells of *Syssomonas* with engulfed starch granules hiding into the starch crystals druse (circles and arrows show life cells of *Syssomonas* floating towards the starch crystals druse). **s**–**u**, **w** Cell aggregations of *Syssomonas* near the bottom of Petri dish. **v** Floating aggregation of flagellated cells. **x** General view of flagellated cell (scanning electron microscopy, SEM). **y** Cyst (SEM). ac, acroneme (pointed tip of flagellum); bc, bacterium; fl, flagellum; fp, filopodium; lb, lobopodium. Scale bars: **a**–**p**, **s**–**w** 10 μm, **r** 45 μm, **x** 3 μm, **y** 2 μm


The organism is characterized by a large variety of life forms including flagellates, amoeboflagellates, amoeboid non-flagellar cells, and spherical cysts (Fig. 1c). The most common stage in the life cycle, a swimming flagellate cell, resembles a typical opisthokont cell, reminiscent of sperm cells of most animals and zoospores of the chytrid fungi. Cells are round to oval and propel themselves with a single, long posterior flagellum (Fig. 2a–c, x). The flagellum is smooth and emerges from the middle-lateral point of the cell, turns back, and always directs backward during swimming. The cell rotates during swimming (Video 1). Flagellar beating can be very fast, which can create the appearance of two flagella. Motile flagellates can suddenly stop and change the direction of movement. The flagellated cells measure 7–14 μm in diameter. The flagellum length is 10–24, rarely 35 μm. Cyst diameter is 5 μm (Fig. 2d, y).

**Additional file 2: Video S1.** Swimming of *Syssomonas multiformis* cell with rotation.


Solitary cells of *Syssomonas* can temporarily attach to the substrate by the anterior part of the cell body. They produce water flow by rapid flagellum beating posteriorly and in that state resemble cells of choanoflagellates or choanocytes from sponges (Fig. 2e, Video 2). Floating flagellated cells can also move to the bottom and transform to amoeboflagellates (Fig. 2j, Video 3) by producing both wide lobopodia and thin short filopodia. Flagellar beating becomes slower and then stops. Amoeboflagellates crawl along the surface using their anterior lobopodia and can take up clusters of bacteria. The organism can lose the flagellum via three different modes: the flagellum may be abruptly discarded from its proximal part of the cell; a stretched flagellum may be retracted into the cell; the flagellum may convolve under the cell body and then retract into the cell as a spiral (Video 4). As a result *Syssomonas* turns into an amoeba (Fig. 2k, l, Video 4). Amoeboid cells produce thin, relatively short filopodia and sometimes have two contractile vacuoles. Amoeboid cells are weakly motile. The transformation of amoeboflagellates and amoebae back to flagellates was also observed.


**Additional file 3: Video S2.** Attached cell of *Syssomonas multiformis* and rapid flagellum beating.



**Additional file 4: Video S3.** Amoeboflagellate stage of *Syssomonas multiformis.* Cells of eukaryotic prey *Parabodo caudatus* are also visible.



**Additional file 5: Video S4.** Loss of flagellum in *Syssomonas multiformis* and transition to amoeba.


Amoeboid cells can also retract their filopodia, become roundish, and transform into a cyst (Fig. 2d, Video 5). Palintomic divisions may occur inside the cyst, and up to 16 (2, 4, 8, or 16) flagellated cells are released as a result (Fig. 2m, Video 6). Division into two cell structures was also observed in culture (Video 7), but it is hard to tell whether a simple binary longitudinal division of a *Syssomonas* cell with retracted flagellum has taken place, or the final stage of a division inside the cyst has been observed.


**Additional file 6: Video S5.** Transformation of amoeba into a cyst in *Syssomonas multiformis*.



**Additional file 7: Video S6.** Palintomic divisions inside the cyst of *Syssomonas multiformis*.



**Additional file 8: Video S7.** Division into two cell structures in *Syssomonas multiformis*.


Floating, flagellated cells containing vesicular structures were observed (Fig. 2n, Additional file 1: Fig. S1E, Video 8); however, the process of formation and the purpose of these vesicles are unknown. After some time, such cells lose their flagellum and transform into vesicular cysts with a thick cover (Fig. 2o). Division inside vesicular cysts was not observed within 10 days of observation. Such structures could represent resting cysts or dying cells containing autophagic vacuoles (the partial destruction of one such cyst was observed after 4 days of observation, see Video 8).


**Additional file 9: Video S8.** Cell and cyst of *Syssomonas multiformis* with vesicular structures inside.


The organism is a predator; it takes up other flagellates (e.g., *Parabodo caudatus* and *Spumella* sp.) which can be smaller, about the same size, or larger than *Syssomonas.* But in contrast to many other eukaryotrophic protists, *Syssomonas* does not possess any extrusive organelles for prey hunting. After initial contact, *Syssomonas* attaches to the prey cell and sucks out their cytoplasm (without ingesting the cell membrane) (Fig. 2f–h, Additional file 1: Fig. S1A-C, Video 9). The organism feeds better on inactive, slow-moving, or dead cells and can also capture intact prey cells and cysts by means unobserved. After attaching to the prey, many other *Syssomonas* cells become attracted to the same prey cell (likely by chemical signaling) and try to attach to it. Joint feeding was observed: several cells of *Syssomonas* can suck out the cytoplasm of the same prey cell together (Fig. 2i, Additional file 1: Fig. S1D, Video 9).


**Additional file 10: Video S9.** Feeding of *Syssomonas multiformis* on eukaryotic prey.


In culture, *Syssomonas* can take up starch granules from rice grains; the granules can be the same size as the cells (Fig. 2p, q). In the presence of *Syssomonas* cells, rice grains in Petri dishes crumble into small fragments and separate granules of starch (Additional file 1: Fig. S2). Cells of *Syssomonas* with engulfed starch granules can hide within the starch crystals druses and lose the flagellum (Fig. 2r, Additional file 1: Fig. S1F). Numerous cysts integrated into the starch matrix were often observed in culture.

The organism can also feed on clusters of bacteria (Video 10) using short pseudopodia. After feeding, *Syssomonas* cells become 2–3 times bigger and a large food vacuole is formed at the posterior end of the cell body (Fig. 2c). In the absence of eukaryotic prey (cultivation on bacteria and/or rice grain/starch only), *Syssomonas* either dies or forms resting cysts. Bacteria alone are not sufficient food for *Syssomonas.*


**Additional file 11: Video S10.** Feeding of *Syssomonas multiformis* on bacteria.


Solitary cells of *Syssomonas* can partially merge and form temporary cell aggregations. They are usually shapeless, observed near the bottom, and consist of about 3–10 flagellated or non-flagellated cells (Fig. 2s–u, Video 11). Another type of aggregation is formed by only flagellated cells with outwards-directed flagella that can float in the water column and resemble the rosette-like colonies of choanoflagellates (Fig. 2v, Video 12). Both types of aggregations break up easily, and it seems that the membranes of such aggregated cells are not fused.


**Additional file 12: Video S11.** Temporary cell aggregations of *Syssomonas multiformis.*



**Additional file 13: Video S12.** Floating rosette-like aggregation of *Syssomonas multiformis.*


However, in rich culture, solitary cells of *Syssomonas* can sometimes merge completely at the bottom of the Petri dish and form syncytium-like (or pseudoplasmodium) structures (it seems that the nuclei do not merge after cell fusion, Fig. 2w). The budding of young flagellated daughter cells from such syncytia was observed (Video 13). Such syncytial structures with budding daughter cells have not been observed in other eukaryotes, to our knowledge, but multinucleated structures arising as a result of multiple cell aggregations or fusions of uninuclear cells are also known in Dictyostelia (Eumycetozoa) and *Copromyxa* (Tubulinea) in the Amoebozoa (sister group of Opisthokonta), as well as in other protists, such as Acrasidae in the Excavata, *Sorogena* in the Alveolata, *Sorodiplophrys* in the Stramenopiles, and *Guttulinopsis* in the Rhizaria [43–47]. Within the opisthokonts, aggregation of amoeboid cells is known in the sorocarpic species *Fonticula alba* (Holomycota) [48]. Transition from filopodial to aggregative stage was also observed in the filasterean *Capsaspora owczarzaki* [49].


**Additional file 14: Video S13.** Syncytium-like structures and budding of young flagellated daughter cells in *Syssomonas multiformis.*


We should also note that a syncytium is not an unusual cell structure in many fungi and animals; for example, most of the cytoplasm of glass sponges (Hexactinellida), the teguments of flatworms, and the skeletal muscles and the placenta of mammals [50, 51] have a syncytial structure.

In *Syssomonas*, the processes of cells merging attract (again, likely by chemical signaling) many other cells of *Syssomonas*, which actively swim near aggregates or syncytium-like structures and try to attach to them. Some of these cells succeed to merge, and the aggregates grow.

All aggregations and syncytial-like structures appear to form by mergers of existing cells in the culture, as opposed to cell division (although, strictly speaking, all cells in the clonal culture are offspring of a single cell of *Syssomonas*).

All of the above-described life forms and cellular changes do not represent well-defined phases of the life cycle of *Syssomonas*, but rather embody temporary transitions of cells in culture which are reversible.

*Syssomonas* grows at room temperature (22 °C) and can survive at temperatures from + 5 to 36 °C. At high temperature (30–35 °C), the prey cells (bodonids) in culture become immobile and roundish; *Syssomonas* actively feeds on these easily accessible cells, multiplies, and produces high biomass. In the absence of live eukaryotic prey, increasing the incubation temperature does not lead to an increase in cell numbers. The cells grow at pH values from 6 to 11. Agitation of culture does not lead to the formation of cell aggregates as was observed in the filasterean *Capsaspora* [49].

### *Syssomonas multiformis* cell ultrastructure

**Fig. 3:**
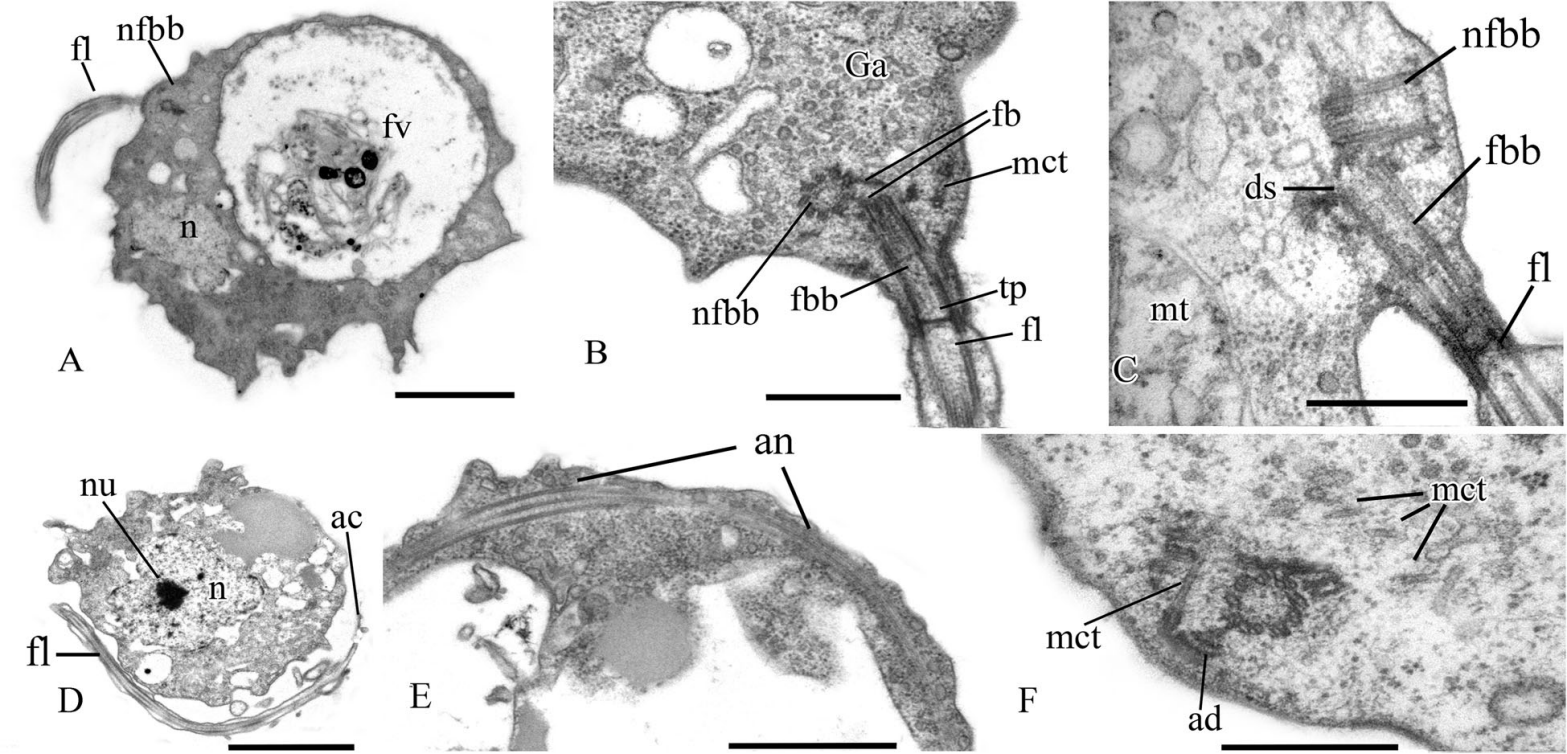
General view and flagellar structure of *Syssomonas multiformis* (transmission electron microscopy, TEM). **a** General view of the cell section with flagellum and large anterior food vacuole (note that ruffled cell outline could be an artifact of fixation). **b**, **c** Arrangement of flagellum and basal bodies and their connection. **d** Twisting of the acronematic flagellum around the cell with nucleus and nucleolus. **e** Retracted flagellum axoneme inside the cell. **f** Basal body of the flagellum and nearest structures. ac, acroneme; ad, arc-like dense structure; an, flagellum axoneme; ds, dense spot; fb, fibril; fbb, flagellar basal body; fl, flagellum; fv, food vacuole; Ga, Golgi apparatus; mct, microtubule; mt, mitochondrion; n, nucleus; nfbb, non-flagellar basal body; nu, nucleolus; tp, transversal plate. Scale bars: **a** 2, **b** 0.5, **c** 0.5, **d** 2, **e** 0.5, **f** 0.5 μm

The cell is naked and surrounded by the plasmalemma. The naked flagellum ends in a short, narrowed tip—the acroneme (Figs. 2x and 3d). A single spiral or other additional elements (e.g., a central filament typical for choanoflagellates) in the transition zone of the flagellum were not observed (Fig. 3b, c). The flagellar axoneme (the central strand of flagellum) has an ordinary structure (9 + 2) in section (not shown). The flagellum can be retracted into the cell (Fig. 3e). A cone-shaped rise at the cell surface around the flagellum base was observed (Fig. 3b, c). The flagellar transition zone contains a transversal plate which is located above the cell surface (Fig. 3b).

**Fig. 4:**
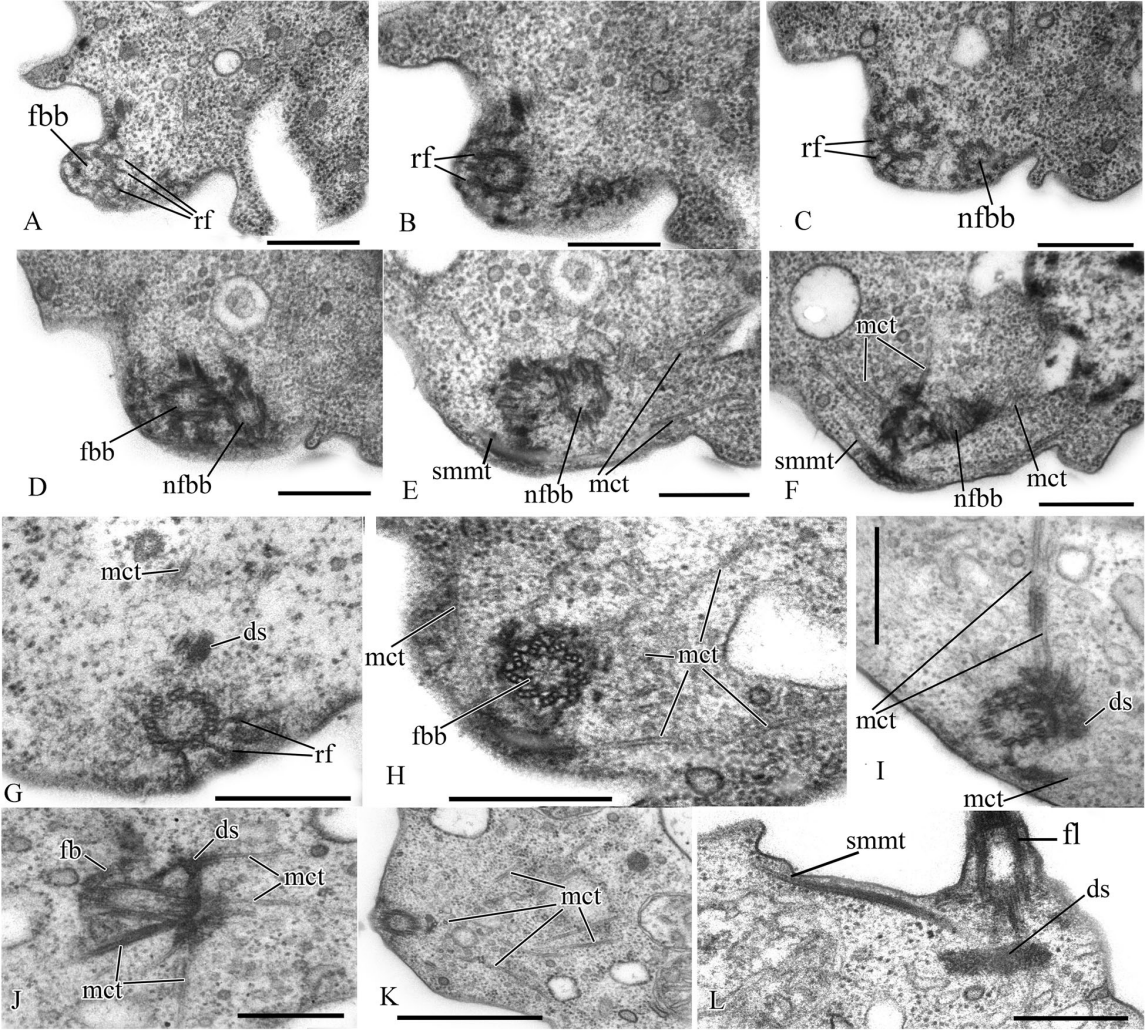
Arrangement of kinetosomes of *Syssomonas multiformis*. **a**–**f** Serial sections (50 nm thickness) of the kinetosomal area, non-flagellar and flagellar basal body with radial fibrils, and submembrane microtubules are visible. **g**–**l** Structures nearby the kinetosomes: microtubules run into the cell, submembrane microtubules begin from the dense spot (**l**) and go below plasmalemma. ds, dense spot; fb, fibril; fbb, flagellar basal body; mct, microtubule; nfbb, non-flagellar basal body; rf, radial fibrils; smmt, submembrane microtubules. Scale bars: **a**–**j**, **l** 0.5, **k** 1 μm

Two basal bodies, one flagellar and one non-flagellar (Fig. 3a–c), lie approximately at a 45–90° angle to each other (Fig. 3b, c). The flagellar root system consists of several elements. Arc-like dense material, representing satellites of the kinetosome, is connected with the flagellar basal body and initiates microtubules which run into the cell (Fig. 3f). Radial fibrils originate from the flagellar basal body (Fig. 4 a–c, g) and resemble transitional fibers. At least two fibrils connect to the basal bodies (Fig. 3b). It can be seen from serial sections that microtubules originate near both basal bodies (Fig. 4a–f). Dense (osmiophilic) spots are situated near the basal bodies, and some of them initiate bundles of microtubules (Fig. 4i, j, l). Microtubules originating from both basal bodies singly or in the form of a fan probably run into the cell (Fig. 3b, Fig. 4f–k). One group of contiguous microtubules begins from the dense spot (Fig. 4l) and goes superficially close to the plasmalemma (Fig. 4e, f, l).

**Fig. 5:**
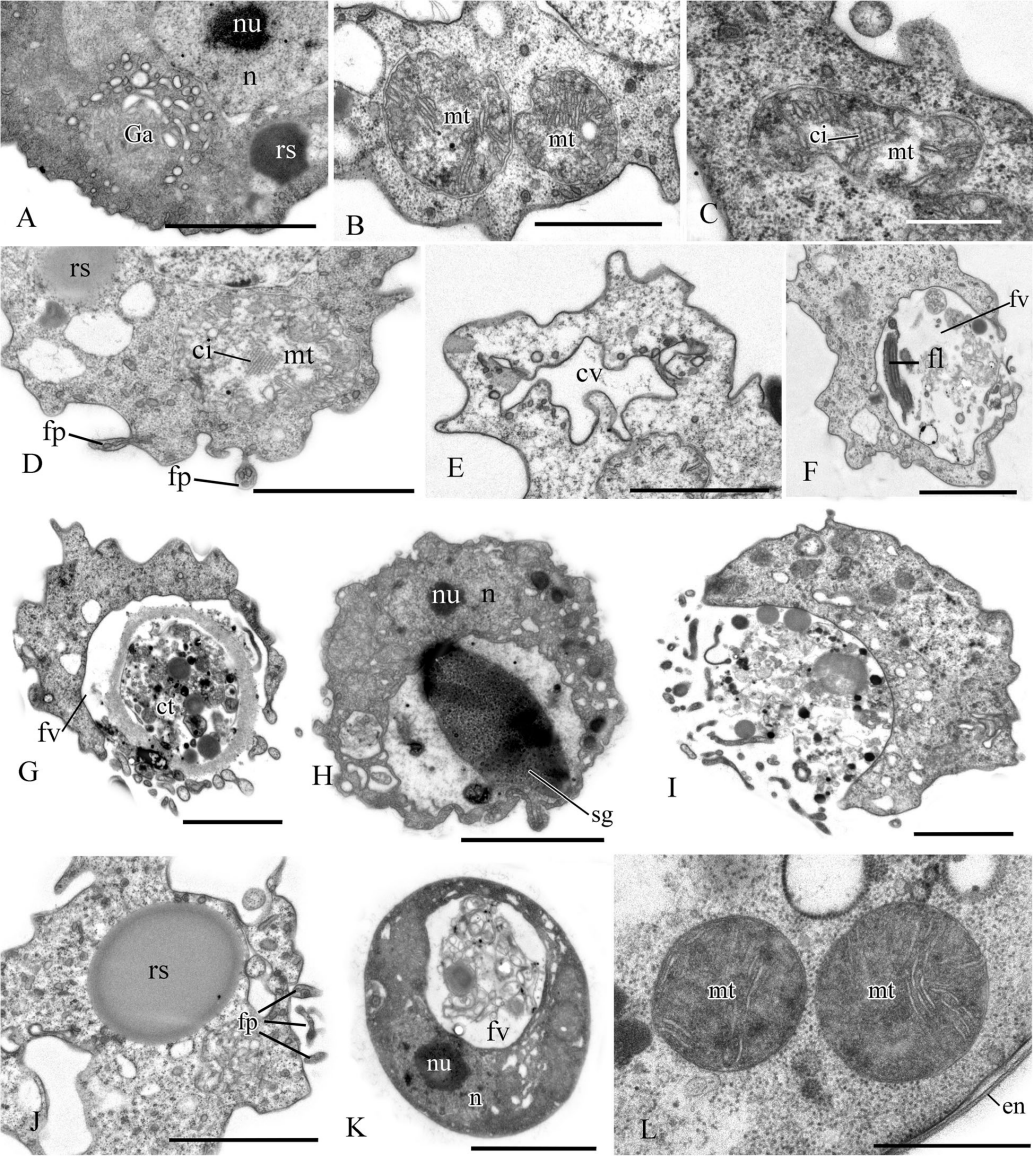
Cell structures and organelles of *Syssomonas multiformis*. **a** Golgi apparatus. **b**–**d** Mitochondria. **e** Contractile vacuole. **f** Food vacuole with remnants of eukaryotic prey (*Parabodo*, flagella, and paraxial rods are seen) at the beginning of exocytosis. **g** Food vacuole containing cyst of *Parabodo* at the beginning of exocytosis. **h** Food vacuole containing starch granule. **i** Exocytosis of food vacuole. **j** Reserve substance and filopodia. **k**, **l** Cysts. ci, crystalloid inclusion; ct, cyst of the prey cell; cv, contractile vacuole; en, cyst envelope; fl, flagellum; fp, filopodia; fv, food vacuole; Ga, Golgi apparatus; mt, mitochondrion; n, nucleus; nu, nucleolus; rs, reserve substance; sg, starch granule. Scale bars: **a** 2, **b** 1, **c** 0.5, **d** 2, **e** 2, **f** 2, **g** 2, **h** 2, **i** 2, **j** 2, **k** 2, **l** 1 μm

The nucleus is 2.6 μm in diameter, has a central nucleolus, and is situated closer to the posterior part of the cell (Fig. 3a, d, Fig. 5h). The Golgi apparatus is of usual structure and is positioned close to the nucleus (Fig. 3b, Fig. 5a). The cell contains several mitochondria with lamellar cristae (Fig. 5b–d). Unusual reticulate or tubular crystal-like structures of unknown nature were observed inside the mitochondria (Fig. 5c, d). A contractile vacuole is situated at the periphery of the cell and is usually surrounded by small vacuoles (Fig. 5e).

A large food vacuole is usually located posteriorly or close to the cell center and contains either remnants of eukaryotic prey, e.g., cells (paraxial flagellar rods are seen) or cysts (fibrous cyst envelope is seen) of *Parabodo caudatus*, or starch granules (Fig. 3a, Fig. 5f–h). Exocytosis occurs on the posterior cell end (Fig. 5i). Storage compounds are represented by roundish (presumably glycolipid) granules 0.8 μm in diameter (Fig. 5a, d, j).

Thin filopodia are located on some parts of cell surface (Fig. 5d, j).

A flagellum or flagellar axoneme, or two kinetosomes, as well as an eccentric nucleus, mitochondria with lamellate cristae and dense matrix, and a food vacuole with remnants of the prey cells, are all visible inside cysts containing dense cytoplasm (Fig. 5k, l).

Extrusive organelles for prey hunting were not observed in any cell type.

### *Pigoraptor vietnamica* morphology and life cycle

**Fig. 6:**
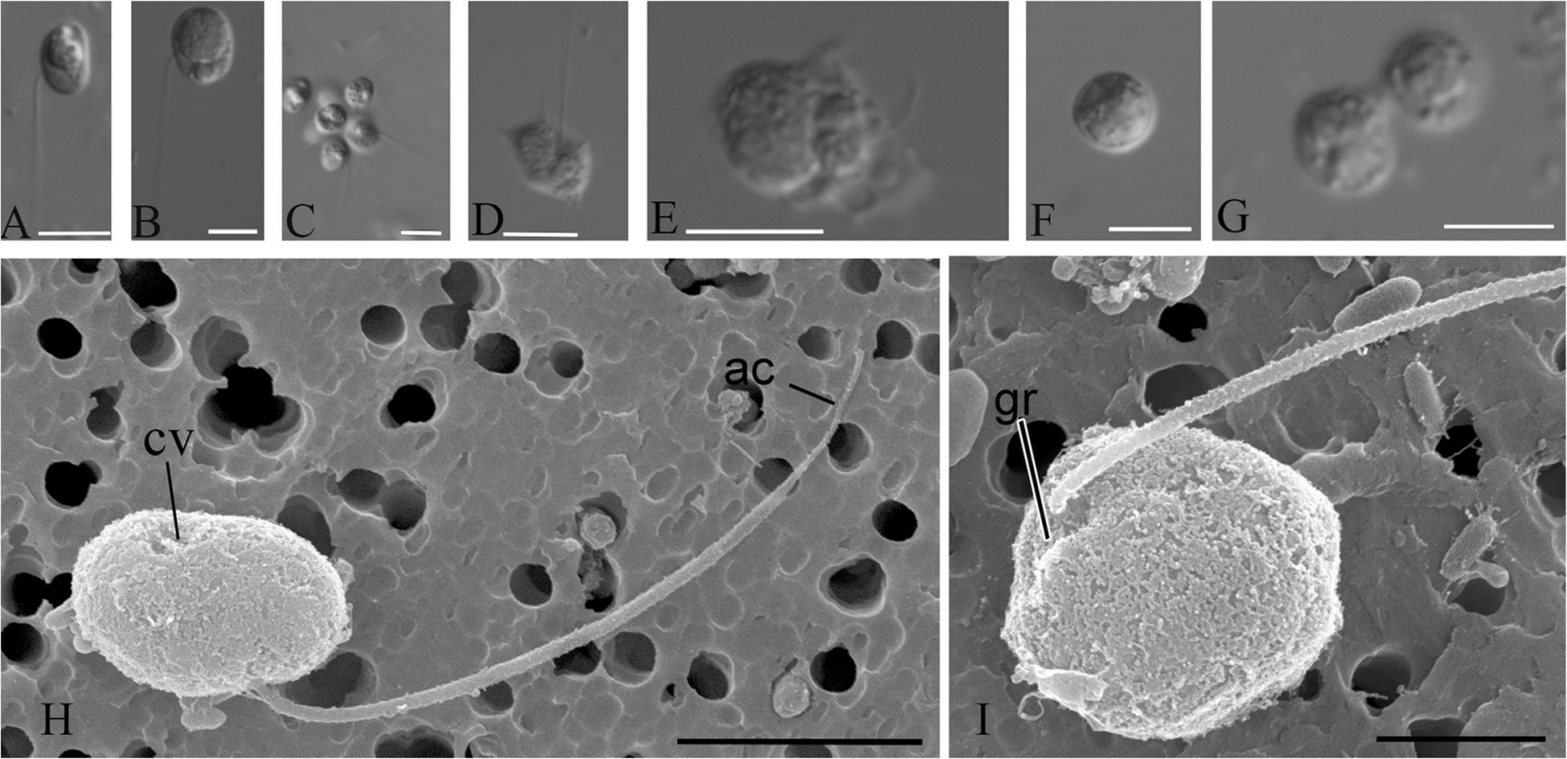
External morphology and life forms of *Pigoraptor vietnamica*. **a**, **b**, **h**, **i** General view of the cell (differential interference contrast, DIC and SEM). **с** Aggregation of flagellated cells. **d** Amoeboflagellate with filopodia. **e** Cell with lobodopia. **f** Cyst. **g** Binary division. ac, acroneme; cv, contractile vacuole; gr, groove. Scale bars: **a**–**g** 10, **h** 4, **i** 2 μm

The uniflagellated, elongated-oval cells are 5–12 μm long (Fig. 6a, b, h, i). The flagellum length is 9–14 μm. Saturated cells with a large food vacuole become roundish. The body plan, movement, feeding, and growth conditions of *Pigoraptor* are identical to *Syssomonas multiformis*, except for the feeding on starch granules, which was not observed in *Pigoraptor*.

The main stage of the life cycle is a swimming flagellated cell, which can form thin long, sometimes branching filopodia that can attach to the substrate (Fig. 1d, Fig. 6d). Wide lobopodia were also observed on some cells (Fig. 6e). Non-flagellate crawling amoebas were not observed. *Pigoraptor* cells can retract the flagellum and become roundish. After several hours, such spherical cells either divide into two daughter cells or turn into cysts (Fig. 6f), which stay intact for a long period. Binary division was observed also inside the cyst (Fig. 6g), resulting in two daughter cells that produce flagella and disperse.

Cells of *Pigoraptor vietnamica* also form easily disintegrating aggregations (Fig. 6c) and feed jointly (Video 14). The adjacent cells can partially merge (likely without membranes fusion) during feeding. These processes also seem to attract many other cells of *Pigoraptor.*


**Additional file 15: Video S14.** Joint feeding of *Pigoraptor vietnamica* on dead cell of *Parabodo caudatus.*


### *Pigoraptor vietnamica* cell ultrastructure

**Fig. 7:**
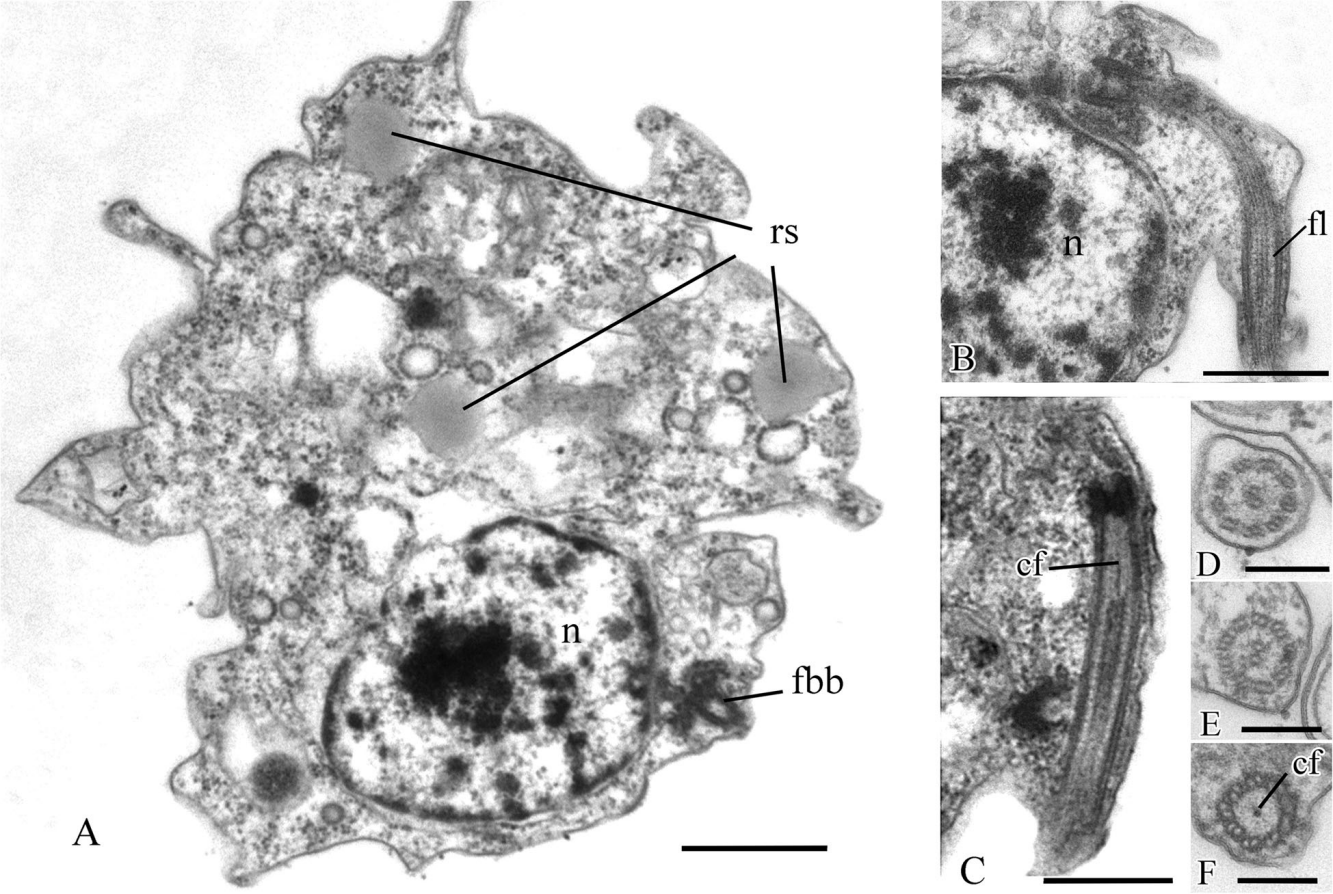
General view and flagellum structure of *Pigoraptor vietnamica*, TEM. **a** Longitudinal cell section. **b**, **c** Longitudinal section of flagellum. **d**–**f** Transverse flagellum sections in transitional area. cf, central filament; fbb, flagellar basal body; fl, flagellum; n, nucleus; rs, reserve substance. Scale bars: **a** 1, **b** 0.5, **c** 0.5, **d**–**f** 0.2 μm

**Fig. 8:**
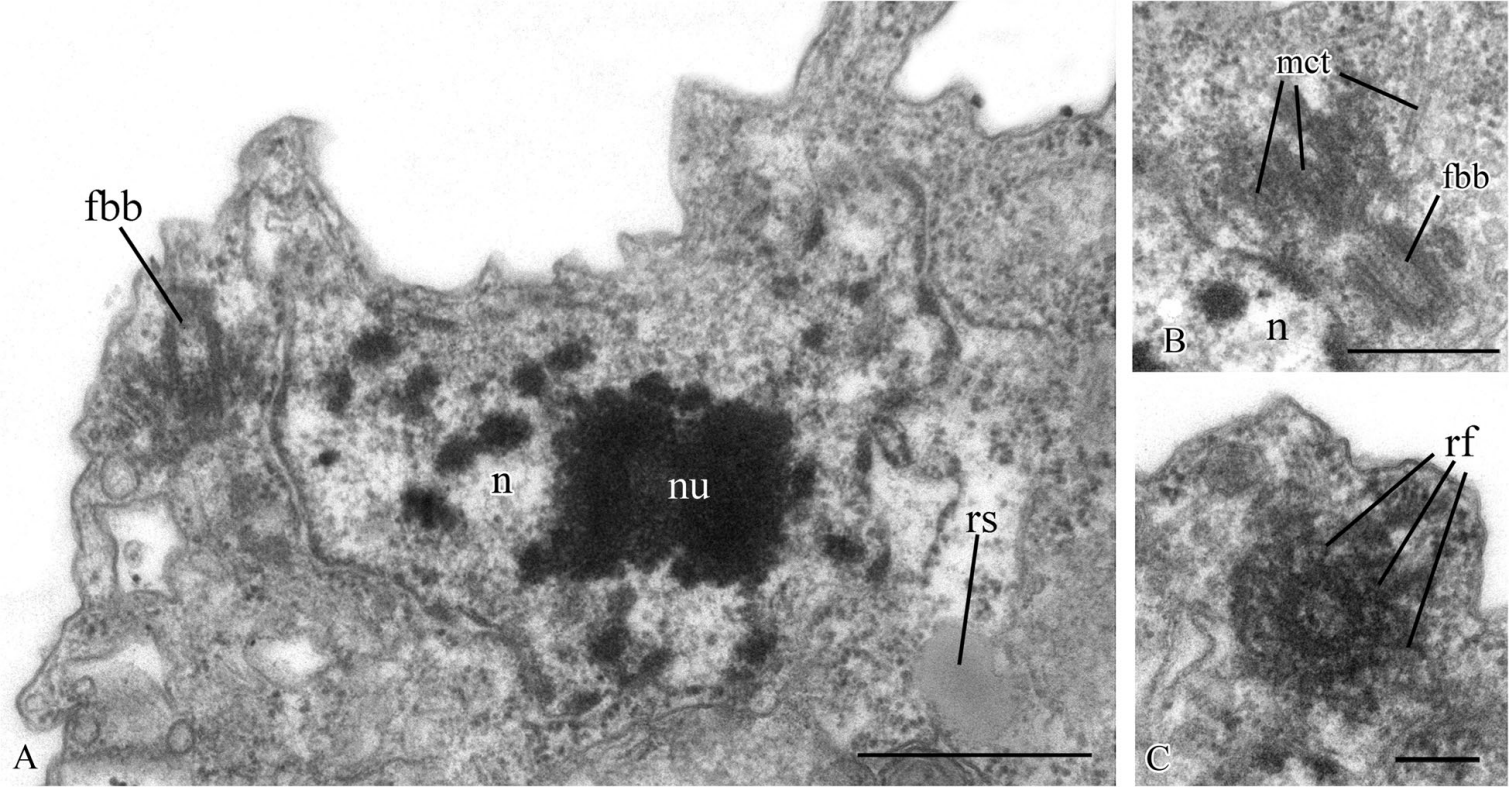
Nucleus and arrangement of basal bodies of *Pigoraptor vietnamica*. **a** Part of the cell containing nucleus and flagellar basal body. **b** Non-flagellar basal body. **c** Flagellar basal body and surrounding structures. fbb, flagellar basal body; mct, microtubule; n, nucleus; nu, nucleolus; rf, radial fibrils; rs, reserve substance. Scale bars: **a** 0.5, **b** 0.5, **c** 0.2 μm

**Fig. 9:**
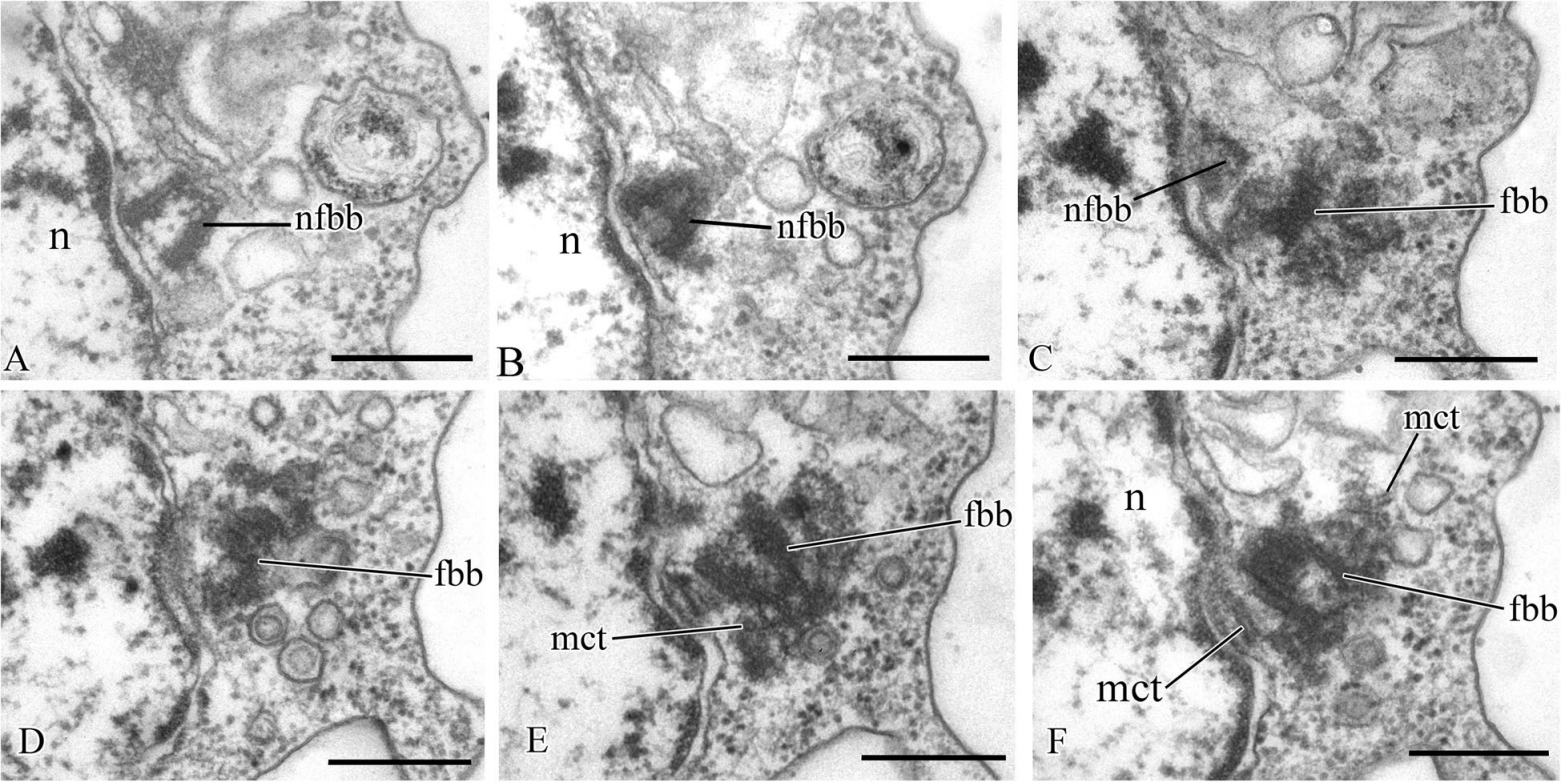
Arrangement of two basal bodies of *Pigoraptor vietnamica* relative to one another. **a**–**f** Serial sections of basal bodies. fbb, flagellar basal body; mct, microtubule; n, nucleus; nfbb, non-flagellar basal body. Scale bars: **a**–**f** 0.5 μm

**Fig. 10:**
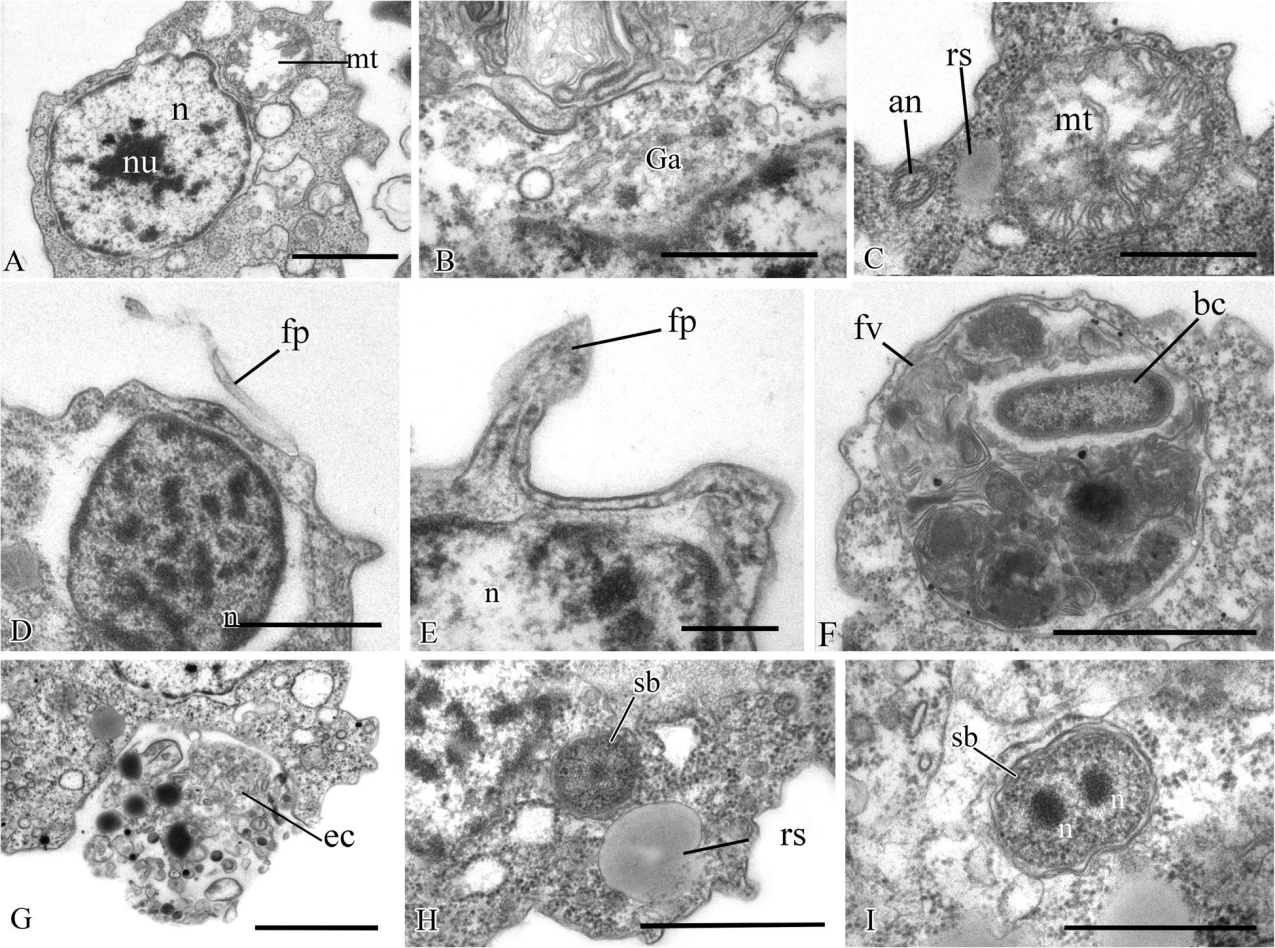
Sections of nucleus and other cell structures of *Pigoraptor vietnamica*. **a** Nucleus. **b** Golgi apparatus. **c** Mitochondrion. **d**, **e** Nucleus and filopodia. **f** Food vacuole. **g** Exocytosis. **h** Reserve substance. **i** Dividing symbiotic bacteria. an, flagellar axoneme; bc, bacterium; ec, ectoproct; fp, filopodium; fv, food vacuole; Ga, Golgi apparatus; mt, mitochondrion; n, nucleus; nu, nucleolus; rs, reserve substance; sb, symbiotic bacteria. Scale bars: **a** 1, **b** 0.5, **c** 0.5, **d** 0.5, **e** 0.2, **f** 1, **g** 1, **h** 1, **i** 0.5 μm

A single, naked flagellum with an acroneme originates from a small lateral groove and directs backward (Fig. 6h, i). The cell is naked and surrounded by the plasmalemma. Two basal bodies, flagellar and non-flagellar, are located near the nucleus, lie approximately at a 90° angle to each other, and are not connected by visible fibrils (Fig. 7a, b; Fig. 8a, b; Fig. 9a–f). The flagellum axoneme has an ordinary structure (9 + 2) in section (Fig. 7d, e). A thin central filament, which connects the central pair of microtubules to the transversal plate, was observed (Fig. 7c, f). The flagellum can be retracted into the cell (Fig. 10c, see axoneme).The flagellar root system is reduced. Radial fibrils arise from the flagellar basal body (Fig. 8c). Microtubules pass near the flagellar basal body (Fig. 8b, Fig. 9e, f). Serial sections show that the non-flagellar basal body does not initiate the formation of microtubules (Fig. 9a, b).

The roundish nucleus is about 1.5 μm in diameter, contains a prominent nucleolus (Fig. 7a, b; Fig. 8a; Fig. 10a, d), and is situated closer to the posterior end of the cell. Chromatin granules (clumps) are scattered within the nucleoplasm. The Golgi apparatus is adjacent to the nucleus (Fig. 10b). Cells contain several mitochondria that possess lamellar cristae (Fig. 10a, c). Rare thin filopodia have been observed on the cell surface (Fig. 10d, e). Cells usually contain one large food vacuole (Fig. 10f), which contains remnants of eukaryotic prey and bacteria. Exocytosis takes place on the anterior cell end (Fig. 10g). Storage compounds are represented by roundish (presumably glycolipid) granules 0.3–0.4 μm in diameter (Fig. 7a, Fig. 8a, Fig. 10c, h). Some cells contain symbiotic bacteria, which are able to divide in the host cytoplasm (Fig. 10h, i). A single contractile vacuole is situated close to the cell surface (not shown on cell sections but visualized by SEM, Fig. 6h).

### *Pigoraptor chileana* morphology and life cycle

**Fig. 11:**
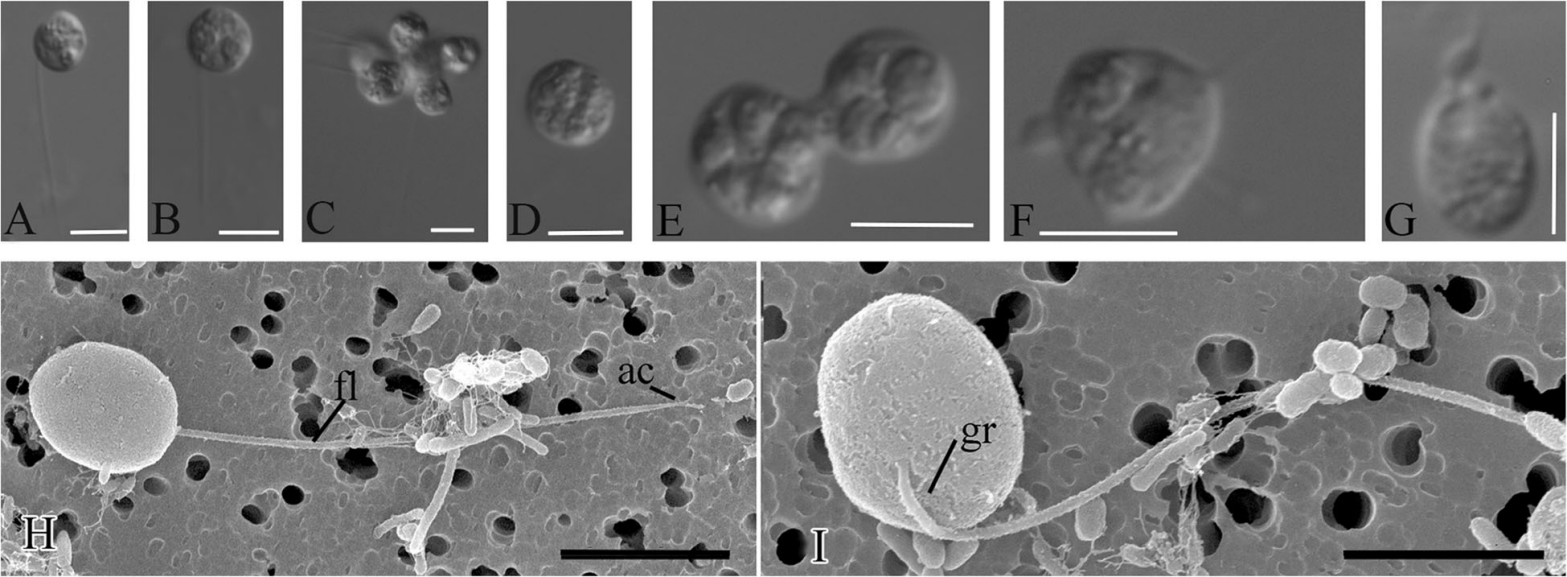
External morphology and life forms of *Pigoraptor chileana*. **a**, **b**, **h**, **i** General view of flagellated cell (DIC and SEM). **с** Cell aggregation. **d** Cyst. **e** Binary division. **f**, **g** Cell with short lobopodia and filipodia. ac, flagella arconeme; gr, groove; fl, flagellum. Scale bars: **a**–**g** 10, **h** 9, **i** 4 μm


The uniflagellated, roundish cells measure 6–14 μm in diameter. The flagellum emerges from a shallow groove and is 8–16 μm in length (Fig. 11a, b, h, i). The flagellum ends with the acroneme. This species is indistinguishable from *Pigoraptor vietnamica* in body plan, movement, feeding, growth conditions*,* joint feeding and aggregation behaviors (Fig. 1d, Fig. 11c, Video 15, 16), encystation (Fig. 11d), and binary division (Fig. 11e), but additionally characterized by the absence of symbiotic bacteria and much reduced capability to produce filopodia and lobopodia (Fig. 11f, g), which are extremely rare in *Pigoraptor chileana*.

**Additional file 16: Video S15.** Joint feeding of *Pigoraptor chileana* on dead cell of *Parabodo caudatus.*



**Additional file 17: Video S16.** Temporary cell aggregation of *Pigoraptor chileana.*


### *Pigoraptor chileana* cell ultrastructure

**Fig. 12:**
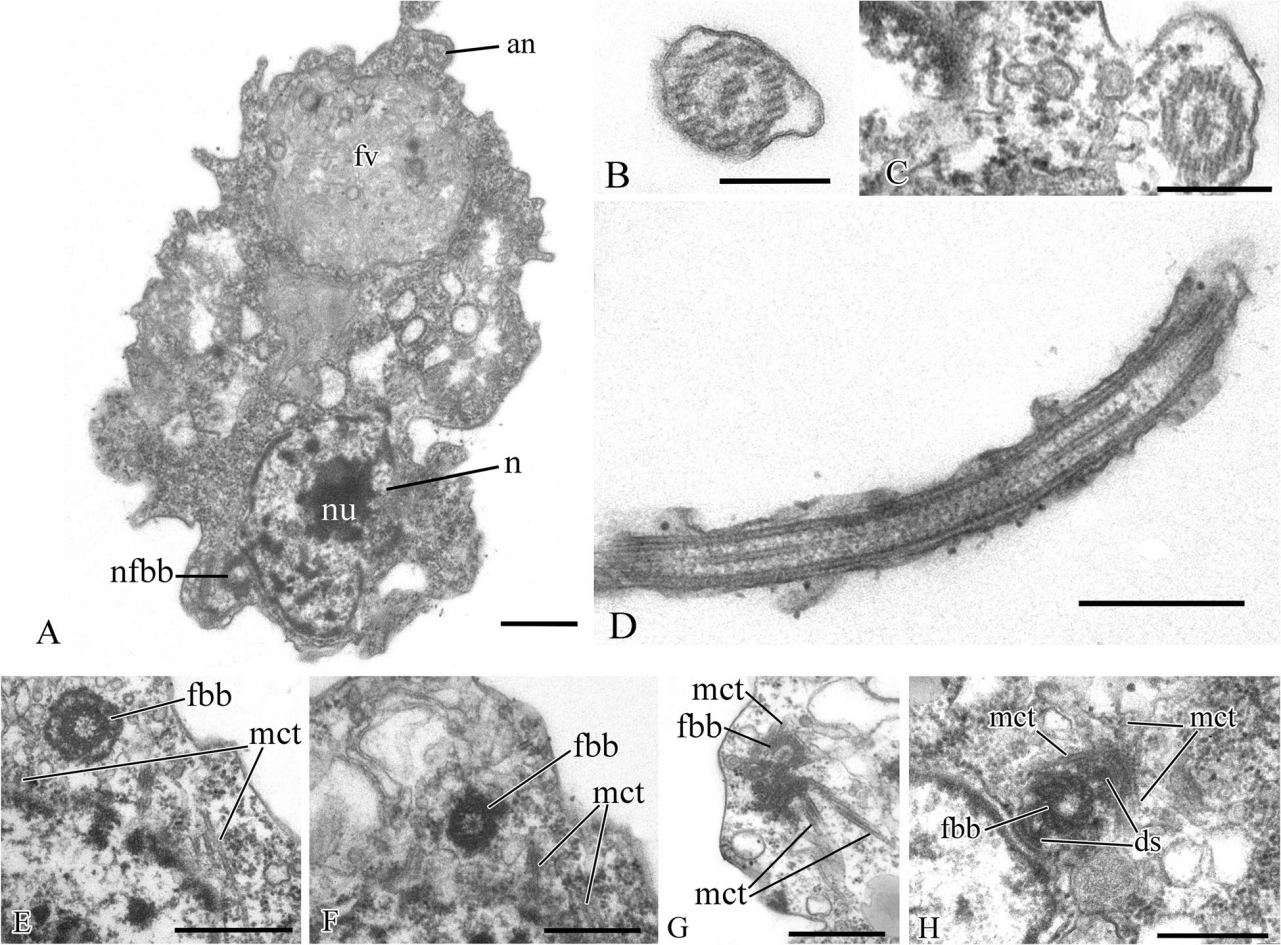
General view, flagellum, and flagella root system of *Pigoraptor chileana*. **a** General view of the cell section. **b**–**d** Flagellum. **e**–**h** Flagellar basal body and surrounding structures. an, axoneme; ds, dense spot; fbb, flagellar basal body; fv, food vacuole; mct, microtubule; n, nucleus; nfbb, non-flagellar basal body; nu, nucleolus. Scale bars: **a** 0.5, **b** 0.2, **c** 0.2, **d** 0.5, **e** 0.5, **f** 0.5, **g** 0.5, **h** 0.5 μm

**Fig. 13:**
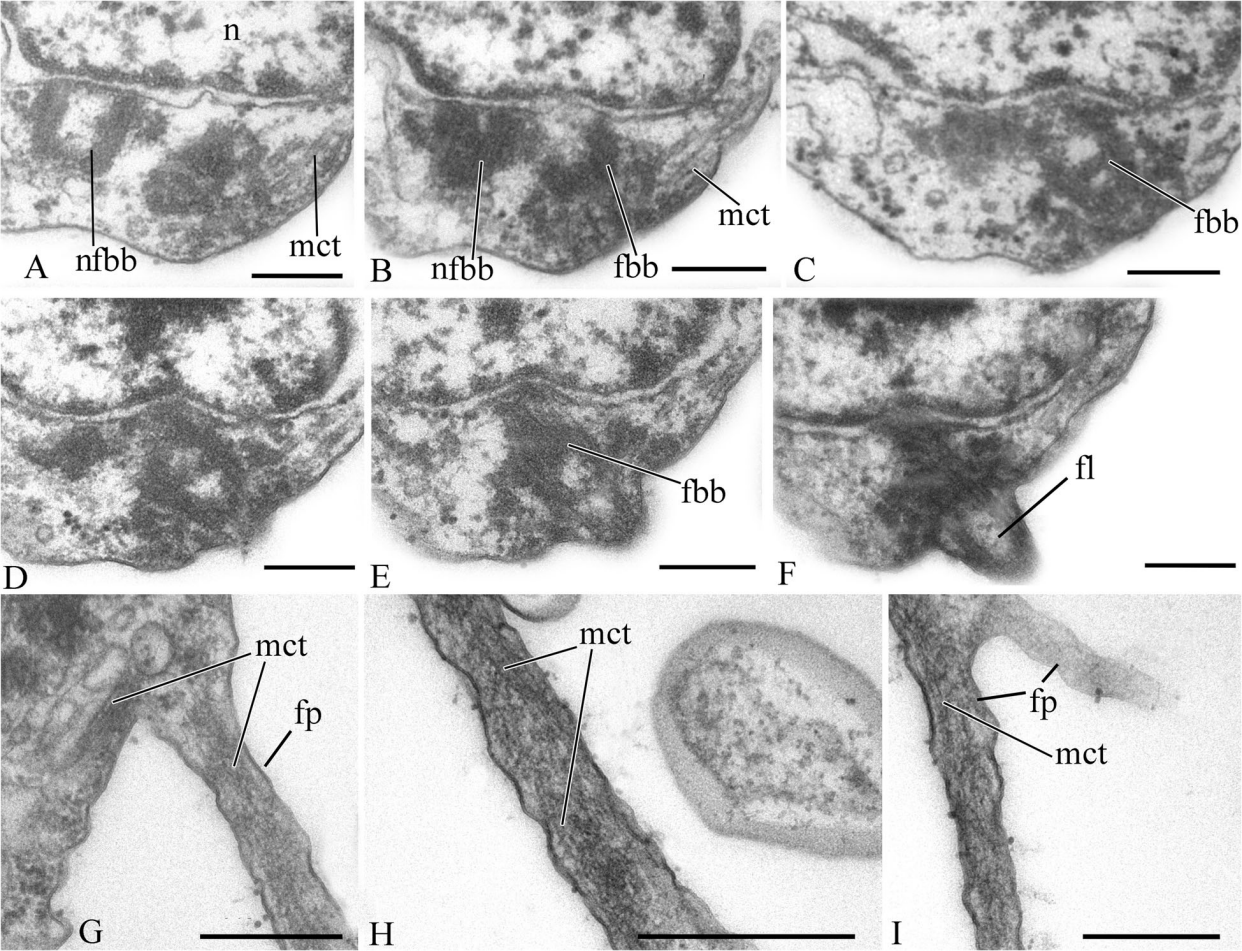
Arrangement of basal bodies and structure of filopodia of *Pigoraptor chileana*. **a**–**f** Serial sections of basal bodies. **g**–**i** Filipodia. fbb, flagellar basal body; fl, flagellum; fp, filopodium; mct, microtubule; n, nucleus; nfbb, non-flagellar basal body. Scale bars: **a**–**f** 0.2, **g**–**i** 0.5 μm

The cell is naked and surrounded by the plasmalemma. The nucleus is positioned close to the posterior cell end (Fig. 12a). The flagellum is naked, and the flagellar axoneme has an ordinary structure (9 + 2) in section (Fig. 12b–d). The flagellum can be retracted into the cell which is visible in some sections (Fig. 12a, c). Flagellar and non-flagellar basal bodies are located near the nucleus (Fig. 12a) and lie approximately at a 60–90° angle to each other (Fig. 13a–f). The flagellar basal body contains a wheel-shaped structure in the proximal part (Fig. 12e, f). Single microtubules and microtubule bundles are situated near this basal body (Fig. 12e–h). Some microtubules arise from dense spots close to the basal body (Fig. 12h).

**Fig. 14:**
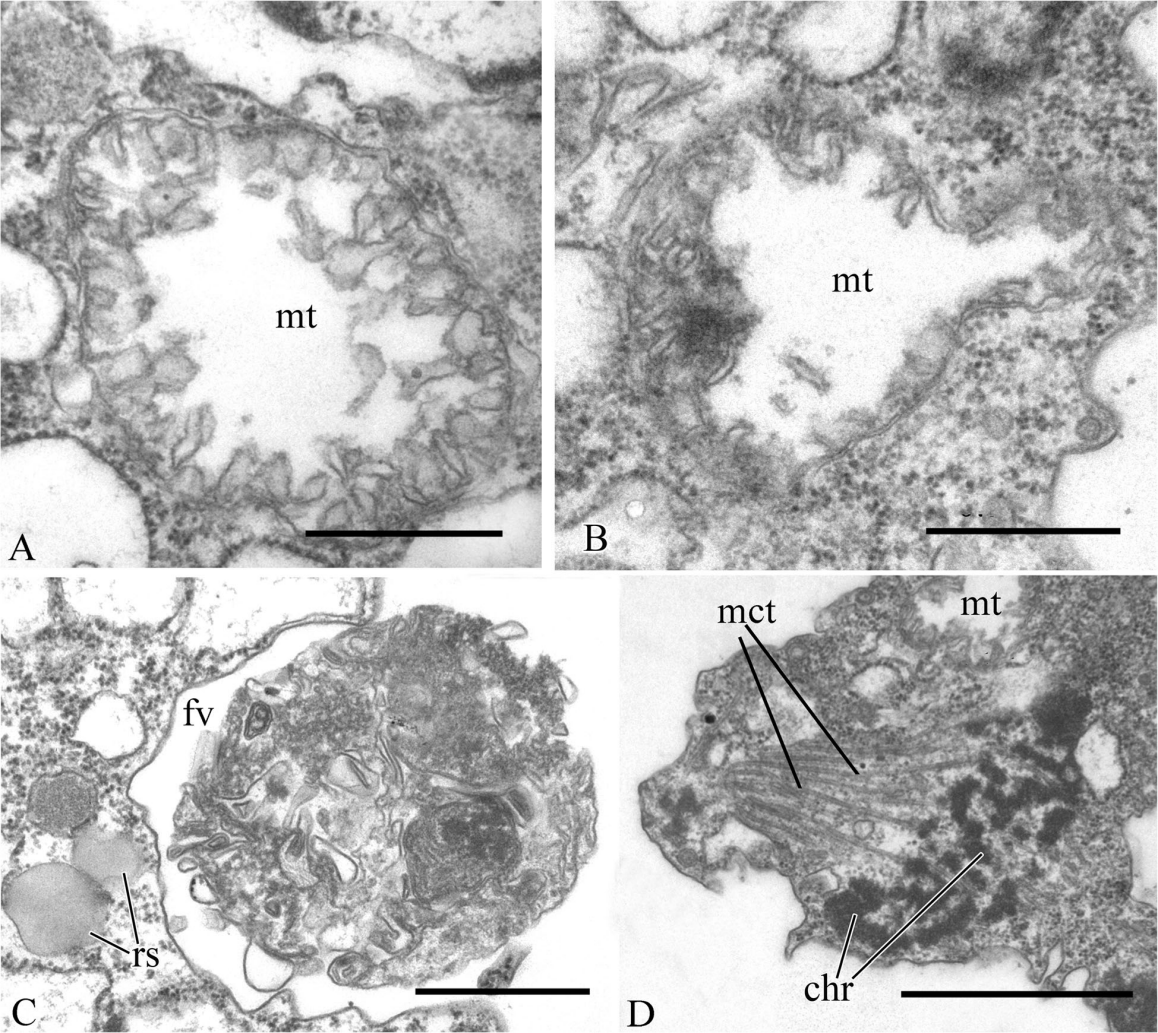
Mitochondria, food vacuole, and nucleus division of *Pigoraptor chileana*. **a**, **b** Mitochondria. **c** Food vacuole and exocytose. **d** Nucleus division in metaphase stage. chr, chromosomes; fv, food vacuole; mct, microtubule; mt, mitochondrion; rs, reserve substance. Scale bars: **a** 0.5, **b** 0.5, **c** 0.5, **d** 1 μm

Rare, thin, sometimes branching filopodia may contain superficially microtubule-like profiles (Fig. 13g–i). The roundish nucleus is about 1.5 μm in diameter and has a central nucleolus (Fig. 12a). Chromatin granules are scattered within the nucleoplasm*.* The Golgi apparatus was not observed. The mitochondria contain lamellar cristae and empty space inside (Fig. 14a, b). Cells usually contain one large food vacuole (Fig. 12a, Fig. 14c), which contains remnants of eukaryotic prey and bacteria. Storage compounds are represented by roundish (presumably glycolipid) granules 0.2–0.4 μm in diameter (Fig. 14c). The single ultrathin section of the dividing cell (possible open orthomitosis) was obtained in metaphase stage (Fig. 14d).

### Key features of novel unicellular opisthokonts and origin of multicellularity in Metazoa

Our understanding of the origin and early evolution of animals has transformed as a result of the study of their most closely related sister groups of unicellular organisms: choanoflagellates, filastereans, and ichthyosporeans [16–18]. Our discovery of previously unknown unicellular Holozoa from freshwater bottom sediments in Vietnam and Chile provides new material for analysis.

#### Eukaryotrophy

A distinctive feature of all three new species is their feeding on eukaryotic prey of similar or larger size, which is unusual (if not unique) for known unicellular Holozoa. While they consume entire prey cells or only the cytoplasmic contents of eukaryotic cells, which resembles the phagocytotic uptake of the contents of *Schistosoma mansoni* sporocysts by the filasterean *Capsaspora owczarzaki* in laboratory conditions [52], they can also feed on clusters of bacteria, resembling the phagocytic uptake of bacteria by choanoflagellates [53]. It is particularly noteworthy that the organisms we discovered do not possess extrusive organelles for paralyzing and immobilizing the prey, which is typical for eukaryovorous protists. Our observations show that prior to absorption, they somehow adhere to the surface of the prey cell. Studies on the choanoflagellate *Monosiga brevicollis* have shown that cadherins, which function as cell-cell adhesion proteins in animals, are located on the microvilli of the feeding collar and colocalize with the actin cytoskeleton [54]. *M. brevicollis* is non-colonial, thus suggesting that cadherins participate in prey capture, not colony formation. In addition, studies of the colonial choanoflagellate *Salpingoeca rosetta* and ichthyosporean *Sphaeroforma arctica* did not indicate a role of the cadherins in colony or “multicellular” epithelial-like structure formation [26, 55], further supporting the notion that cadherins do not play a role in cell-cell adhesion in choanoflagellates and ichthyosporeans, and perhaps also did not in the unicellular ancestor of animals [41]. Also, non-cadherin-based epithelial structure is present in slime molds [56].

In the case of the unicellular predators *Syssomonas* and *Pigoraptor*, adherence to a large and actively moving prey seems to be crucial for feeding and important for survival. Interestingly, the *Syssomonas* transcriptome does not include cadherin genes, but it does express C-type lectins (carbohydrate-binding proteins performing various functions in animals, including intercellular interaction, immune response, and apoptosis). The opposite is seen in *Pigoraptor*, where cadherin domain-containing transcripts were found but no C-type lectins [37].

The presence of eukaryotrophy as a type of feeding within both filastereans and Pluriformea (although the second representative, *Corallochytrium*, is an osmotroph) suggests that predation could be or have once been widespread among unicellular relatives of animals, and perhaps that the ancestor of Metazoa was able to feed on prey much larger than bacteria. The “joint feeding” we observed many times in cultures of *Syssomonas* or *Pigoraptor*, including the behavior where cells are attracted to the large prey by other predators already feeding on it, is probably mediated by chemical signaling of the initially attached predator cell. The newly arriving cells also adhere to the plasmalemma of the prey, partially merging with each other and sucking out the contents of the large prey cell together (Fig. 2 i, Video 9, 14, 15). The merging of predator cells during feeding is quite unusual and may represent a new factor to consider in the emergence of aggregated multicellularity. In addition, putative chemical signaling to attract other cells of its species is observed during the formation of syncytial structures in these species. In this context, alpha- and beta-integrins and other components of the so-called integrin adhesome, which are responsible for interaction with the extracellular matrix and the transmission of various intercellular signals, were found in the transcriptomes of all three studied species [37].

#### Starch breakdown by *Syssomonas*

An interesting phenomenon was observed in clonal cultures of *Syssomonas*, where the predator can completely engulf starch granules of the same size as the cell, also mediating the rapid destruction of rice grains into smaller fragments and individual starch crystals (Additional file 1: Fig. S2). It is possible that *Syssomonas* secretes hydrolytic enzymes that provide near-membrane extracellular digestion. The appearance of extracellular digestion is considered to be a major step in animal evolution [57], since it is central to the breakdown of many organic molecules when combined with the direct absorption of nutrient monomers by the gut epithelia using transmembrane transport in animals [58].

This ability of *Syssomonas* to feed on starch is likely promoted by the expression of numerous enzymes that are putatively involved in starch breakdown (several putative α-amylases and α-glucosidases, a glycogen debranching enzyme, and a glycogen phosphorylase) (Table 1). For example, *Syssomonas* has five distinct putative α-amylases, one of which was not found in any other Holozoa present in our database (Table 1). Similarly, one of the four putative α-glucosidases in *S. multiformis* seems to be specific to this lineage, and possibly the Filasterea, within the Holozoa. While α-amylases and α-glucosidases are able to hydrolyze α-1,4-linked glycosidic linkages, mobilization of the starch molecule at the α-1,6 glycosidic bonds at branch points requires the activity of debranching enzymes. A possible candidate for the catalysis of this reaction is a conserved glycogen debranching enzyme in *S. multiformis*, orthologous to the human *AGL* gene (Table 1). Additionally, we identified a transcript for a glycogen phosphorylase (orthologous to the human *PYGB*, *PYGL*, and *PYGM* genes), an enzyme involved in the degradation of large branched glycan polymers.

**Table 1:** Transcripts for putative starch-degrading enzymes in *S. multiformis* and the presence/absence of corresponding orthologs in other unicellular holozoan lineages and Metazoa

	*S. multiformis*	Ichthyosporea	Filasterea	Choanoflagellates	Metazoa
α-Amylases (PF00128)					
1	Colp12_sorted@267, Colp12_sorted@268, Colp12_sorted@269	*Pirum_gemmata*@Unigene16826_1*	Ministeria_vibrans@37663/4*	Craspedida_sp_A TCC_50635@246766*	NA
2	Colp12_sorted@19603	NA	NA	NA	NA
3	Colp12_sorted@18987, Colp12_sorted@16467, Colp12_sorted@17178	*Pirum_gemmata*@Unigene17488_1	*Ministeria_vibrans*@28157	*Salpingoeca_rosetta*@00083T0	*Amphimedon_queenslandica*@15720304
4 = paralog to 3	Colp12_sorted@8715	See 3	See 3	See 3	See 3
5 (*SLC3A1*)	Colp12_sorted@10150	NA	NA	*Salpingoeca_rosetta*@08827T0	*Homo_sapiens*@ENSP00000260649
α-Glucosidases (PF01055)					
1	Colp12_sorted@9290, Colp12_sorted@22295	*Pirum_gemmata*@Unigene@34248_1*	*Ministeria_vibrans@34861*	*Salpingoeca_rosetta*@00005T0*	NA
2	Colp12_sorted@1339, Colp12_sorted@1341	*Creolimax_fragrantissima*@8464T1	*Capsaspora_owczarzaki*@04827T0	NA	*Amphimedon_queenslandica*@15725226
3 = paralog to 2	Colp12_sorted@9196, Colp12_sorted@14409, Colp12_sorted@18957, Colp12_sorted@21457, Colp12_sorted@31517	See 2	See 2	NA	See 2
4. (*GANAB/GANC*)	Colp12_sorted@13767	*Creolimax_fragrantissima*@4769T1	*Capsaspora_owczarzaki*@00308T0	*Salpingoeca_rosetta*@11360T0	*Homo_sapiens*@ENSP00000326227/ENSP00000349053/ENSP00000340466
Glycogen phosphorylase (*PYGB/PYGL/PYGM*)					
1	Colp12_sorted@1564	*Creolimax_fragrantissima*@2598T1	*Capsaspora_owczarzaki*@07395T0	*Salpingoeca_rosetta*@08531T0	*Homo_sapiens*@ENSP00000216392/ENSP00000216962/ENSP00000164139
Glycogen debranching enzyme (*AGL*)					
1	Colp12_sorted@14615	*Creolimax_fragrantissima*@4755T1	*Capsaspora_owczarzaki*@06406T0	*Salpingoeca_rosetta*@10237T0	*Homo_sapiens*@ENSP00000355106

Accession numbers for *S. multiformis* are from the corresponding TransDecoder output files as described in Hehenberger et al. [37]; sequence identifiers for all other taxa correspond to the deposited alignments/phylogenies. Pfam domains used to identify candidates in *S. multiformis* and/or annotated direct human orthologous genes are indicated in brackets in column 1. NA, no direct orthologs recovered in our dataset; transcript identifiers in italic, short fragment(s) of one lineage representative

*Unclear whether sequences are direct orthologs or paralogs to *S. multiformis* (unresolved tree)

Our observations also show that in the presence of starch in culture, *Syssomonas* can form resting stages of unidentified genesis, which tend to adhere to each other and to starch grains.

#### Structural features

*Syssomonas* and *Pigoraptor* both display a broad morphological plasticity: all three species have a flagellar stage, form pseudopodia and cysts, and can form aggregations of several cells. *Syssomonas multiformis* also has an amoeboid non-flagellar stage. The dominant life form of all three species in culture is the uniflagellar swimming cell. Interestingly, amoeboid and pseudopodial life forms were detected in cultures only after 2 years of cultivation and observation, suggesting they may be extremely rare in nature. Overall, the morphological differences between cells of the same type of the two genera, *Syssomonas* and *Pigoraptor*, are few and subtle. Given these genera are distantly related within the tree of Holozoa in all current phylogenomic reconstructions, it is interesting to speculate that they may be the result of morphostasis and by extension retain features resembling those of an ancestral state of extant holozoan lineages. Although other unknown lineages of unicellular holozoans undoubtly exist and their morphology remains to be investigated, we propose such lineages will likely also possess a similar morphological plasticity, with flagellated and pseudopodial stages, and characteristics overall similar to *Syssomonas* and *Pigoraptor*. It has been established that single cells of *Syssomonas* and *Pigoraptor* can temporarily attach themselves to the substrate (Fig. 1c, d) and, by beating their flagellum, can create water currents to putatively attract food particles, similar to choanoflagellates and sponge choanocytes (Fig. 2e, Video 2), although such behavior could be analogous. Choanocytes and choanoflagellates possess, in addition to the flagellum, a collar consisting of cytoplasmic outgrowths reinforced with actin filaments (microvilli) that serve to capture bacterial prey. The thin filopodia that are observed on the cell surface of all three *Syssomonas* and *Pigoraptor* species may thus be homologous to collar microvilli. But this will require further evidence in form of homologous proteins in these structures or evidence of their function in *Syssomonas* and *Pigoraptor*. While the filopodia of *Syssomonas* have no obvious structural contents, the outgrowths of *Pigoraptor* sometimes contain microtubular-like profiles. Cross-sections of these structures were not obtained, but they may represent parallel microfilaments such as recently found in the filopodial arms of *Ministeria vibrans* [25]. The organization of the *Ministeria* filopodial arms resembles the microvilli of choanoflagellates, which have stable bundles of microfilaments at their base. It has been proposed previously that the ancestor of Filozoa (Filasterea + Choanoflagellida + Metazoa) probably had already developed filose tentacles, which have aggregated into a collar in the common ancestor of choanoflagellates/sponges [24], and that microvilli were present in the common ancestor of Filozoa [25].

A single, posterior flagellum is the defining characteristic of opisthokonts [34]. However, the flagellum has not yet been found in all known Opisthokonta lineages. Torruella et al. [19] have found several proteins corresponding to key components of the flagellum in *Corallochytrium* and the filose amoeba *Ministeria vibrans*, which have been considered to lack flagella. These authors have shown that the stalk used by *Ministeria* to attach to the substrate is a modified flagellum. Recently, morphological observations on another strain of *Ministeria vibrans* (strain L27 [25]) revealed that this strain lacks the stalk for substrate attachment, but possesses a typical flagellum that projects forward and beats at attached to the substrate cells (see Fig. 2h and Video S1 in [25]). The authors concluded that the filasterean ancestor possessed a flagellum, which was subsequently lost in *Capsaspora owczarzaki*. In the case of *Corallochytrium limacisporum*, it was suggested that it has a cryptic flagellate stage in its life cycle [19], as has been proposed for other eukaryotes (*Aureococcus* and *Ostreococcus*, for instance) based on their genome sequences [59]. Therefore, the flagellate stage could have been the one morphological trait uniting *Corallochytrium* and *Syssomonas* within “Pluriformea.” Interestingly, the ancestor of ichthyosporeans probably also had a flagellum, which is preserved in the Dermocystida (at the stage of zoospores), but was again lost in the Ichthyophonida. However, some membrane-decorated vesicles with short flagellum-like strands were visualized by TEM on cell sections in ichtyophonid *Sphaeroforma sirkka* and *S. napiecek* [60].

The central filament of the flagellum, which connects the central pair of microtubules with the transversal plate in *Pigoraptor* (Fig. 7c, f), is also noteworthy, since this character was previously known only in the choanoflagellates [61] and was considered a unique feature for this lineage. The cone-shaped elevation of the surface membrane around the base of the flagellum in *Syssomonas* (Fig. 3b, c) is also typical for choanoflagellates (see Fig. 5a, b in [61]).

One interesting ultrastructural peculiarity is the unusual reticulate or tubular structures in *Syssomonas* mitochondria which were not observed in other eukaryotes we are aware of.

Another noteworthy feature is the presence of symbiotic bacteria in the cells of *Pigoraptor vietnamica*. Symbiotic bacteria are common in both vertebrate and invertebrate animals including placozoan *Trichoplax* [62] and fungi [63]. Bacterial endosymbionts are also known in unicellular protists from all eukaryotic supergroups and perform a multitude of new biochemical functions in the host [64, 65]. But as far as we know, there are only two documented cases of opisthokont protists with prokaryotic symbionts: nucleariid amoebae (sister lineage of fungi) with several groups of Proteobacteria and the choanoflagellate *Codosiga balthica*, which harbored two different endosymbiotic bacteria inside the cytoplasm [66, 67]. Future investigations of the intracellular bacteria in *Pigoraptor* will be essential to understanding the role of prokaryotic symbionts in the biology and cell functions of unicellular relatives of animals.

#### Origins of multicellularity

As mentioned above, numerous theories about the origin of Metazoa exist. One of the first and widely accepted evolutionary theories on the origin of animals is the Gastrea theory of Ernst Haeckel [68]. Haeckel suggested that the first step in the evolution of multicellularity in animals was the formation of a hollow ball, the walls of which consisted of undifferentiated flagellated cells, which he called Blastea. This was followed by gastrulation, where the ball invaginated, leading to the primary cellular differentiation into ecto- and endoderm. Modern variants of this, for example, the сhoanoblastea theory, which highlights the similarity between Haeckel’s Blastea and the choanoflagellate colony [57], are the most common and influential explanations for the origin of multicellular animals. An important assumption of these theories is that cell differentiation took place only *after* multicellularity arose, suggesting that animals originated from a single cell type, such as choanoflagellate-like colony-forming ancestor [41], which is supported by the possible homology between sponge choanocytes and choanoflagellates [69], and consistent with the Haeckel-Muller Biogenetic Law that ontogenesis recapitulates phylogenesis [70].

There are, however, some basic differences between sponges and choanoflagellates in how their collar and flagella interact, suggesting that choanocyte/choanoflagellate similarity might be superficial and that specific homology cannot be automatically assumed (see [71] for details). Indeed, recent ultrastructural studies on sponge choanocytes and choanoflagellates show that they are fundamentally different in many respects (see [72–74] for details), some of which are among the very few ultrastructural systems in eukaryotic cells considered sufficiently conservative to indicate phylogenetic relationships [75–77]. For example, specific features of the flagellar apparatus of the sponge *Ephydatia fluviatilis* are more similar to zoospores of chytrids than to choanoflagellates [78]. Overall, the conclusion that sponges (and by extension all Metazoa) descend directly from a single-celled organism similar to choanoflagellates is not unambiguously supported by ultrastructure, as sometimes assumed [73].

Recent comparative transcriptomic analysis also argues against a direct link between sponge choanocytes and choanoflagellates, suggesting instead that the first animal cell had the ability to transition between multiple states in a manner similar to modern transdifferentiating stem cells [79]. This is more consistent with an alternative class of ideas very different from the Gastrea hypothesis, which suggest the presence of diverse life forms and complex life cycles were the initial step in the origin of animal multicellularity. Or in other words, that *differentiation preceded multicellularity* and the origin of the blastula. Unicellular relatives of animals have now long been known to contain a variety of genes homologous to those involved in cell adhesion, differentiation, development, and signal transduction in Metazoa. Some of them were once considered to be unique to animals (e.g., transcription factors T-box and Rel/NF-kappa B, Crumbs protein, integrin beta) as they were absent in choanoflagellates (the closest relatives of Metazoa), but later, they were found to be present in other unicellular Holozoa [24, 31, 80]. Now, homologs of most genes controlling the development of animals, their cell differentiation, an cell-cell and cell-matrix adhesion are known in various lineages of unicellular organisms [18, 81–83], all suggesting that genetic programs of cellular differentiation and adhesion arose relatively early in the evolution of opisthokonts and before the emergence of multicellularity [15, 24, 31, 81, 84].

One specific hypothesis focusing on cell differentiation preceding the formation of colonies is the “synzoospore hypothesis” [85, 86], and see [31] for details. In brief, three types of cell cycle are present in the ontogenesis of multicellular animals: monotomy (alternate phases of cell growth and division of somatic cells), hypertrophic growth (in female sex cells), and palintomy (the egg undergoes a series of consecutive divisions). Zakhvatkin noted that some protists alternate between different types of life cycle, and suggested that the unicellular ancestor of Metazoa already had differentiated cells as a result of such a complex life cycle. The life cycle complexity, in turn, results from the fact that monotomic cells are usually sedentary, or at least less mobile, and can change their phenotype (from flagellated to amoeboid, etc.) depending on the environment; the process of palintomy is necessary for the formation of morphologically identical dispersal cells (spores or zoospores). These dispersal cells remain attached to each other, forming a primary flagellated larva—the synzoospore or blastula (cited from [31]).

The synzoospore hypothesis is consistent with recent observations of complex life cycles in unicellular opisthokonts possessing cellular differentiation, the presence of sedentary trophic phases, and a tendency to aggregation, as seen in choanoflagellates [53, 87–90], filastereans [49], *Corallochytrium* [28], ichthyosporeans [17, 84, 91], chytridiomycetes [92], and nucleariid amoebae [93]. According to this theory, multicellularity in animals arose through the temporal integration of various types of cells, which were already present in different parts of the life cycle. The hypothetical ancestor of animals in this model would thus already have genetic programs for cell differentiation (including cadherins, integrins, tyrosine kinases).

Developing the synzoospore hypothesis further, Mikhailov et al. [31] proposed an evolutionary mechanism of “transition from temporal to spatial cell differentiation” to explain the emergence of multicellular animals. In this model, the ancestor of Metazoa was a sedentary colonial protist filter-feeder with colonies formed by cells of different types, which arose because filtration efficiency is significantly enhanced through the cooperation of cells of different types. Dispersal cells produced by the sedentary stage, the zoospores, remained attached together in early metazoans (which increased survivability) as a synzoospore to form a primary larva, the blastula. Development of a whole colony from such a multicellular larva occurred through the *differentiation of genetically identical* zoospore cells. This was critical for the maintenance of long-term cell adhesion and thus emergence of true multicellularity, as opposite to temporary colonies and aggregations composed of genetically heterogeneous cells. The authors suggest that the dispersal stages of the sedentary trophic body—primary blastula-like larvae—acquired adaptations to the predatory lifestyle, which triggered the development of primary intestine, muscular, and nervous systems [31].

In this view, the origin of multicellularity was a *transition from temporal to spatiotemporal cell differentiation* [41]. A bacteriotroph with differentiated, temporally separated cell types became spatially integrated, existing simultaneously but in different parts of a now multicellular conglomerate with different cell types carrying out different functions. From this starting point, further diversification could proceed straightforwardly by expansion of gene regulation networks and signaling pathways.

Genomics alone is not sufficient to distinguish these different models, because morphology, life cycle, and structural features are all relevant and central. All three novel species of unicellular Holozoa have life histories that are consistent with major elements of the synzoospore model (see Fig. 3a in [31] and Fig. 5a, b in [41]). Specifically, these organisms have complex life histories characterized by a variety of forms: flagellates, amoebae, amoeboflagellates, and cysts. All three species have the tendency to form aggregations. *Syssomonas* possesses both clonal and aggregative multicellular stages, as predicted for the ancestor of animals. Moreover, the formation of aggregations can be associated with adhering cysts, but also by active feeding on large eukaryotic prey, which also leads to hypertrophic cell growth (described as proliferative stage in [41]) with a subsequent phase of palintomic division (in *Syssomonas*). All these characters are predicted by the synzoospore model. Interestingly, feeding on large eukaryotic prey appears to be a trigger for several key behaviors, including the formation and development of aggregates (e.g., joint feeding) and clonal multicellularity (e.g., hypertrophic growth followed by palintomy). This is not generally regarded as a key factor in the origin of multicellularity in ancestors of Metazoa, but we suggest this should be considered. It is an interesting parallel that morphological and developmental changes (e.g., colony formation) in choanoflagellates can be also triggered by prey, although bacterial [94–96]. The functional basis for these changes requires further study, as does the relationship between the life cycle, morphology, formation of multicellular structures, and impacts of environmental change.

## Conclusions

As we acquire more information about the biology of known unicellular relatives of animals and, equally importantly, describe diverse new species of unicellular Holozoa, models for the evolutionary histories of specific characteristics that contributed to the emergence of multicellularity in animals can be evaluated more meaningfully. *Syssomonas* and *Pigoraptor* are characterized by complex life cycles, the formation of multicellular aggregations, and an unusual diet for single-celled opisthokonts (partial cell fusion and joint sucking of large eukaryotic prey), all of these features providing new insights into the origin of multicellularity in Metazoa.

Genome and transcriptome analyses of unicellular relatives of animals have shown that genes encoding proteins for cellular signaling and adhesion, as well as genes for embryonic development of multicellular organisms, arose before the emergence of multicellular animals [24, 37, 81, 84]. While these genes likely have different functions in protists than in animals, they nevertheless probably relate to the ability to recognize the cells of their own species, prey, or organic molecules and contribute to the formation of multicellular aggregations, thus increasing the organism’s ability to adapt to environmental change. As we learn more about the natural history and behavior of these organisms, the importance of these processes becomes even more clear. The phylogenetic distribution of unicellular holozoans with complex life cycles suggests that the ancestor of Metazoa probably formed cells of various types that could aggregate and had molecular mechanisms of cell differentiation and adhesion related to those processes. We suggest that this supports the conclusion that cellular differentiation arose before the emergence of multicellularity.

The feeding modes of the ancestral metazoan may also have been more complex than previously thought, including not only bacterial prey, but also larger eukaryotic cells and organic structures. Indeed, the ability to feed on large eukaryotic prey could have been a powerful trigger in the formation and development of both aggregative and clonal multicellular stages that played important roles in the emergence of multicellularity in animals. Lastly, we wish to point out that other new and deep lineages of opisthokonts undoubtedly exist that have not yet been described, and each of these will play an important role in the development of hypotheses on the origin of multicellular animals in future.

## Methods

Novel unicellular opisthokont predators were found in freshwater biotopes in Vietnam and Chile. *Syssomonas multiformis* (clone Colp-12) was obtained from the sample of freshwater pool (11° 23′ 08.0″ N, 107° 21′ 44.9″ E; *T* = 39 °C; pH = 7.18; DO (ppm) = 0.64; conductivity (μS/cm) = 281; TDS (ppm) = 140), Tà Lài, Cát Tiên National Park, Dong Nai Province, Socialist Republic of Vietnam, on April 29, 2013. *Pigoraptor vietnamica* (clone Opistho-1) was obtained from freshwater Lake Dak Minh, silty sand on the littoral (12° 54′ 50″ N, 107° 48′ 26″ E; *T* = 27 °C; pH = 7.03; DO (ppm) = 7.43; conductivity (μS/cm) = 109; TDS (ppm) = 54), Dak Lak Province, Socialist Republic of Vietnam, on March 26, 2015. *Pigoraptor chileana* (clone Opistho-2) was obtained from the bottom sediments of freshwater temporary water body (submerged meadow, 54° 02′ 29.7″ S, 68° 55′ 18.3″ W; *T* = 16.5 °C; pH = 6.62; conductivity (μS/cm) = 141; TDS (ppm) = 72) near the Lake Lago Blanca, Tierra del Fuego, Chile, on November 4, 2015.

The samples were examined on the third, sixth, and ninth days of incubation in accordance with the methods described previously [97]. Following isolation by glass micropipette, freshwater clones Colp-12, Opistho-1, and Opistho-2 were propagated on the bodonid *Parabodo caudatus* (strain BAS-1, IBIW RAS) grown in Pratt’s medium or spring water (Aqua Minerale, PepsiCo, Moscow Region, Russia, or PC Natural Spring Water, President’s Choice, Toronto, Canada) by using the bacterium *Pseudomonas fluorescens* as food [98]. The clone Colp-12 was perished after 5 years of cultivation. The clones Opistho-1 and Opistho-2 are stored in the “Live culture collection of free-living amoebae, heterotrophic flagellates and heliozoans” at the Institute for Biology of Inland Waters, Russian Academy of Science.

Observations of live cells were carried out in the laboratory conditions at 22 °C in the clonal cultures. Studied species were able to survive at 5–40 °C temperatures, pH 6–11, and tolerate salinity increasing up to 4‰. The variation of temperature, pH, and cultivation medium does not result in appearance of additional morphological forms or increasing of frequency of occurrence of certain (e.g., amoeboid) life forms. The agitation of the cultures (up to 1400 rpm) does not lead to the formation of cell aggregations. Clone Colp-12 was also able to grow on stramenopile prey *Spumella* sp. (clone OF-40 from “Live culture collection of free-living amoebae, heterotrophic flagellates and heliozoans” at the Institute for Biology of Inland Waters, Russian Academy of Science). Light microscopy observations were made by using the Zeiss Axio Scope A.1 equipped with a DIC contrast water immersion objective (× 63). The images were taken with the AVT HORN MC-1009/S analog video camera and directly digitized by using the Behold TV 409 FM tuner. Cells with engulfed starch granules were inspected by epifluorescence microscopy after DAPI staining using the Zeiss Axioplan 2 Imaging microscope.

For transmission electron microscopy (TEM), cells were centrifuged, fixed at 1 °C for 15–60 min in a cocktail of 0.6% glutaraldehyde and 2% OsO_4_ (final concentration) prepared using a 0.1-M cacodylate buffer (pH 7.2). Fixed cells were dehydrated in alcohol and acetone series (30, 50, 70, 96, and 100%, 20 min in each step). Afterward, the cells were embedded in a mixture of Araldite and Epon (Luft, 1961). Ultrathin sections (50 nm) were prepared with an Leica EM UC6 ultramicrotome (*Leica Microsystems*, Germany) and observed by using the JEM 1011 transmission electron microscope (JEOL, Japan).

For scanning electron microscopy (SEM), cells from exponential growth phase were fixed as for TEM but only for 10 min at 22 °C and gently drawn onto a polycarbonate filter (diameter 24 mm, pores 0.8 μm). Following the filtration, the specimens were taken through a graded ethanol dehydration and acetone and finally put into a chamber of a critical point device for drying. Then, dry filters with fixed specimens were mounted on aluminum stubs, coated with gold-palladium, and observed with a JSM-6510LV scanning electron microscope (JEOL, Japan).

Analysis of enzymes involved in starch breakdown was based on transcriptomic data obtained as described earlier [37]. To identify candidates putatively involved in starch breakdown, we used the results of a previous hmmscan analysis of *S. multiformis* [37] to search for Pfam domains present in enzymes/enzyme families naturally involved in starch degradation (as described in 10.1016/j.sbi.2016.07.006), such as α-amylases (PF00128), glycoside hydrolase families containing α-glucosidases (PF02056, PF01055, PF03200, PF10566), α-glucan water dikinase 1 (*GWD1*, PF01326), phosphoglucan phosphatase (*DSP4*, PF00782, and PF16561), disproportionating enzymes (PF02446), and pullulanases (PF17967). Additionally, we submitted the *S. multiformis* sorted transcriptome to the KEGG Automatic Annotation Server (KAAS) [99] for functional annotation and investigated the output for transcripts involved in starch metabolism. All candidates were further investigated by BLASTp search [100] against GenBank, by domain analysis using InterProScan (doi:10.1093/bioinformatics/btu031) and by phylogenetic reconstruction. For phylogenetic reconstruction, the candidates were used as queries in a BLASTp search (*e* value threshold 1e−5) against a comprehensive custom database containing representatives of all major eukaryotic groups, including opisthokonts (with a focus on unicellular holozoan lineages, including several choanoflagellates, filastereans, and ichthyosporeans (including *Pigoraptor* sp.) as well as *S. multiformis*—see 10.1016/j.cub.2017.06.006 for data sources), apusomonads, amoebozoans, discobids, archaeplastids, cryptophytes, haptophytes, dinoflagellates, chrompodellids, apicomplexans, ciliates, stramenopiles, and rhizarians, as well as RefSeq data from all bacterial phyla at NCBI (https://www.ncbi.nlm.nih.gov/, last accessed December 2017). The database was subjected to CD-HIT with a similarity threshold of 85% to reduce redundant sequences [101]. Results from blast searches were parsed for hits with a minimum query coverage of 50% and *e* values of less than 1e−5. The number of bacterial hits was restrained to 20 hits per phylum (for FCB group, most classes of Proteobacteria, PVC group, Spirochaetes, Actinobacteria, Cyanobacteria (unranked), and Firmicutes) or 10 per phylum (remaining bacterial phyla) as defined by NCBI taxonomy. Parsed hits were aligned with MAFFT v. 7.212, using the –auto option; poorly aligned regions were eliminated using trimAl v.1.2 with a gap threshold of 80% [102, 103]. Maximum likelihood tree reconstructions were then performed with FastTree v. 2.1.7 using the default options [104]. Phylogenies with overlapping taxa were consolidated by combining the parsed hits of the corresponding queries, removing duplicates and repeating the alignment, trimming, and tree reconstruction steps as described above. All phylogenies were manually investigated, and obvious contaminations removed from the underlying alignment. For the final tree reconstruction, the cleaned, unaligned sequences were then subjected to filtering with PREQUAL using the default options (https://www.ncbi.nlm.nih.gov/pubmed/29868763) to remove non-homologous residues introduced by poor-quality sequences, followed by alignment with MAFFT G-INS-i using the VSM option (--unalignlevel 0.6) (https://www.ncbi.nlm.nih.gov/pubmed/27153688) to control overalignment. After trimming ambiguously aligned sites with trimAl v. 1.2 (-gt 0.8), sequences with less than 50% of the alignment length were removed. Final trees were calculated with IQ-TREE v. 1.6.5 (https://www.ncbi.nlm.nih.gov/pubmed/25371430), using the -mset option to restrict model selection to LG for ModelFinder after initial model testing without any restrictions selected the LG substitution model for all alignments (https://www.ncbi.nlm.nih.gov/pubmed/28481363), while branch support was assessed with 1000 ultrafast bootstrap replicates (https://www.ncbi.nlm.nih.gov/pubmed/29077904). The raw tree files in newick, colored trees with taxon information (and accession numbers where available) in pdf format, and underlying trimmed alignments have been deposited to figshare repository [105] doi: 10.6084/m9.figshare.11914491.

## Supplementary information

**Additional file 1: Fig. S1.** A-C – *Syssomonas multiformis* sucks out the cytoplasm of the prey; D – three cells of *Syssomonas* (*s*) suck out the cytoplasm of the same prey cell together, other *Syssomonas* cells (arrows) become attracted and swim to the same prey cell; E – unusual flagellated cell of *Syssomonas* containing vesicular structures; F – cells of *Syssomonas* with engulfed starch granules swim to the starch crystals druse and hide within the starch crystals. **Fig. S2.** Rice grain destruction in Petri dish with Pratt medium and presence of the cells of *Parabodo caudatus* (p rey) only (A) and *Syssomonas multiformis* (B) after 9 days of incubation.

## Data Availability

Videos of morphology, life cycle, and feeding of novel predatory unicellular relatives of animals are available on figshare [105]. The raw tree files in newick, colored trees with taxon information (and accession numbers where available) in pdf format, and trimmed alignments have been deposited to the same figshare deposition [105] 10.6084/m9.figshare.11914491.
