# Supplementary figures and images for: Insights into the origin of metazoan multicellularity from predatory unicellular relatives of animals
Source: BMC Biol. 2020 Apr 9;18:39. doi: 10.1186/s12915-020-0762-1 (PMC7147346; doi:10.1186/s12915-020-0762-1)

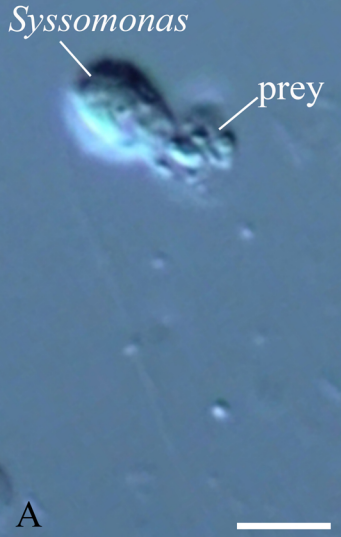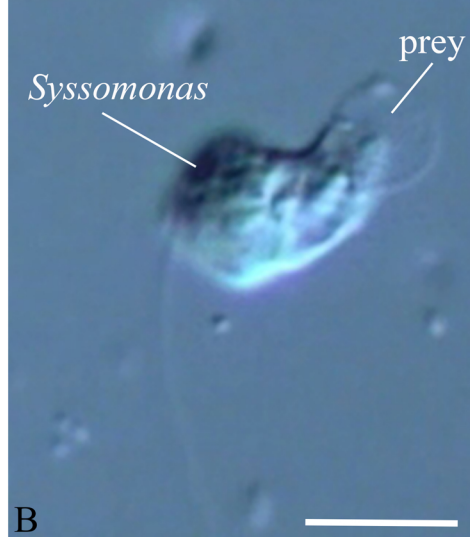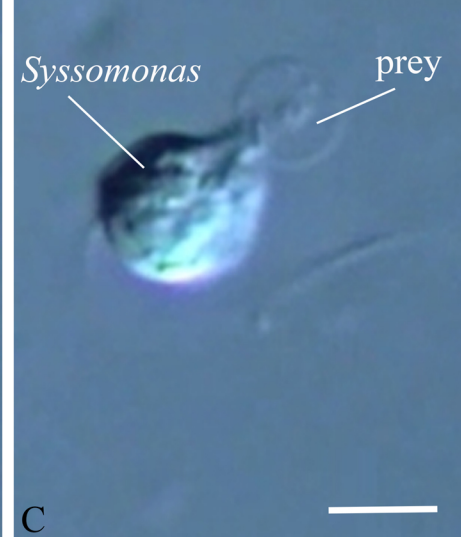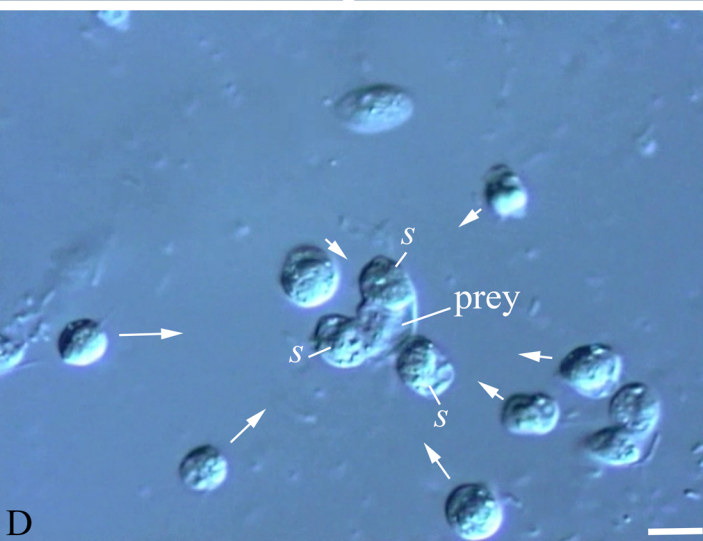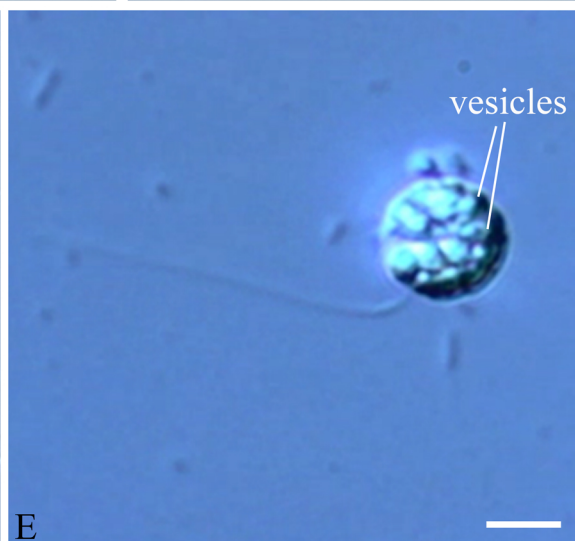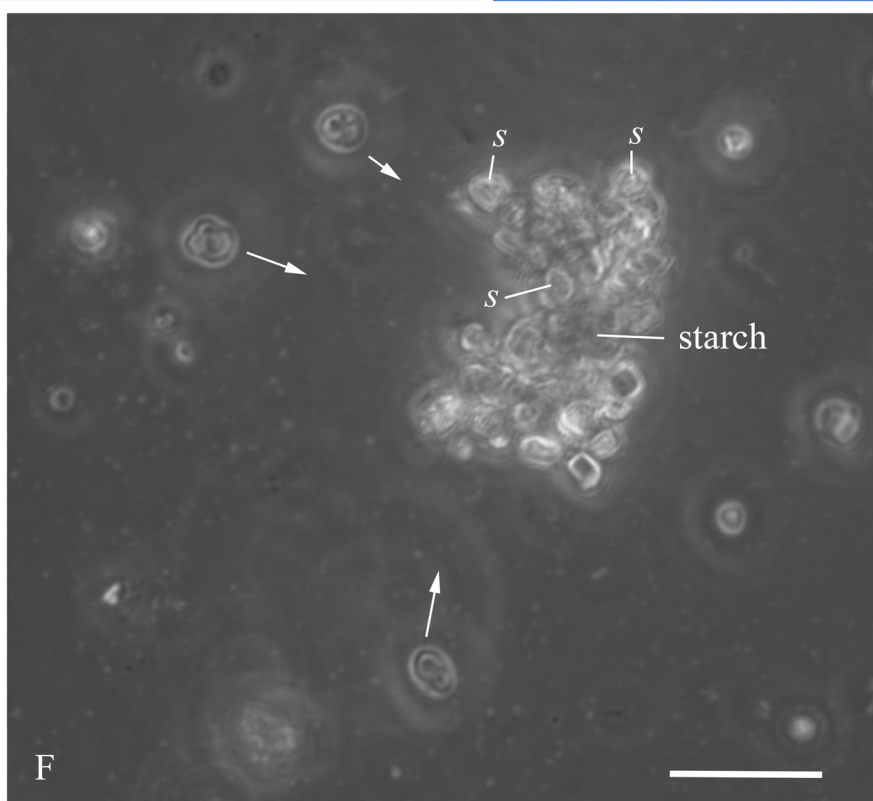

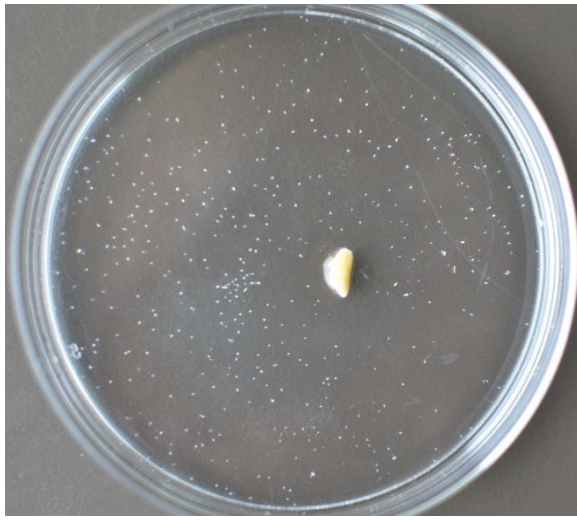

**A**

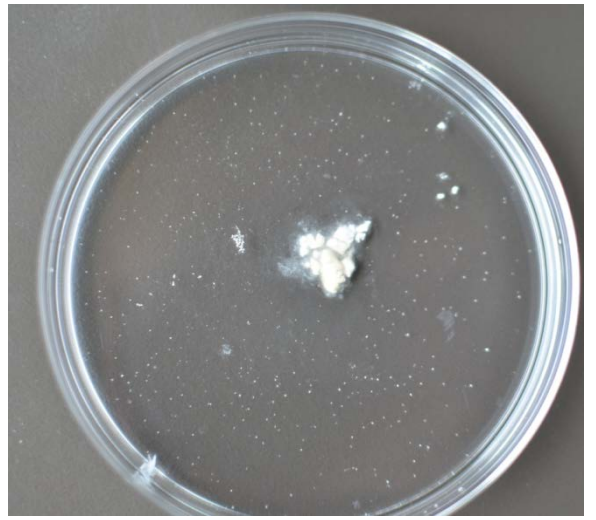

**B**

Supplement: Supplementary file 17 — Additional file 1: Fig. S1. A-C – Syssomonas multiformis sucks out the cytoplasm of the prey; D – three cells of Syssomonas (s) suck out the cytoplasm of the same prey cell together, other Syssomonas cells (arrows) become attracted and swim to the same prey cell; E – unusual flagellated cell of Syssomonas containing vesicular structures; F – cells of Syssomonas with engulfed starch granules swim to the starch crystals druse and hide within the starch crystals. Fig. S2. Rice grain destruction in Petri dish with Pratt medium and presence of the cells of Parabodo caudatus (p rey) only (A) and Syssomonas multiformis (B) after 9 days of incubation. [file 12915_2020_762_MOESM1_ESM.pdf]
